# Integrative analysis of miRNA–mRNA network in high altitude retinopathy by bioinformatics analysis

**DOI:** 10.1042/BSR20200776

**Published:** 2021-01-14

**Authors:** Tong Su, Chufeng Gu, Deji Draga, Chuandi Zhou, Thashi Lhamo, Zhi Zheng, Qinghua Qiu

**Affiliations:** 1 Department of Ophthalmology, Shanghai General Hospital, Shanghai Jiao Tong University School of Medicine, Shanghai, P.R. China; 2 National Clinical Research Center for Eye Diseases; Shanghai Key Laboratory of Ocular Fundus Diseases; Shanghai Engineering Center for Visual Science and Photomedicine; Shanghai Engineering Center for Precise Diagnosis and Treatment of Eye Diseases, Shanghai, P.R. China; 3 Department of Ophthalmology, Shigatse People’s Hospital, Shigatse, Xizang, P.R. China

**Keywords:** Bioinformatics analysis, Differentially expressed genes, High-altitude retinopathy, MiRNA-mRNA network

## Abstract

High-altitude retinopathy (HAR) is an ocular manifestation of acute oxygen deficiency at high altitudes. Although the pathophysiology of HAR has been revealed by many studies in recent years, the molecular mechanism is not yet clear. Our study aimed to systematically identify the genes and microRNA (miRNA) and explore the potential biomarkers associated with HAR by integrated bioinformatics analysis. The mRNA and miRNA expression profiles were obtained from the Gene Expression Omnibus database. We performed Gene Ontology functional annotations and Kyoto Encyclopedia of Genes and Genomes pathway analysis. Potential target gene analysis and miRNA–mRNA network analysis were also conducted. Quantitative RT-PCR (qRT-PCR) was used to validate the results of the bioinformatics analysis. Through a series of bioinformatics analyses and experiments, we selected 16 differentially expressed miRNAs (DE-miRNAs) and 157 differentially expressed genes related to acute mountain sickness (AMS) and constructed a miRNA–mRNA network containing 240 relationship pairs. The hub genes were filtered from the protein-protein interaction network: IL7R, FOS, IL10, FCGR2A, DDX3X, CDK1, BCL11B and HNRNPH1, which were all down-regulated in the AMS group. Then, nine up-regulated DE-miRNAs and eight hub genes were verified by qRT-PCR in our hypoxia-induced HAR cell model. The expression of miR-3177-3p, miR-369-3p, miR-603, miR-495, miR-4791, miR-424-5p, FOS, IL10 and IL7R was consistent with our bioinformatics results. In conclusion, FOS, IL10, IL-7R and 7 DE-miRNAs may participate in the development of HAR. Our findings will contribute to the identification of biomarkers and promote the effective prevention and treatment of HAR in the future.

## Introduction

High-altitude retinopathy (HAR) is one clinical entity of acute high-altitude illness (AAI), which also includes acute mountain sickness (AMS), high-altitude pulmonary edema (HAPE) and high-altitude cerebral edema (HACE) [1,2]. As retinopathy is caused by the inability to adapt to acute oxygen deficiency at high altitudes, HAR mainly manifests as leakage of the peripheral retinal vessels, retinal hemorrhages, tortuous retinal vessels, optic disc edema and macular edema [3,4]. With the development of the economy and tourism in the plateau area, the incidence of HAR is up to 79% and is increasing [5]. However, the pathogenesis of HAR remains unclear, and there is a lack of effective prevention and treatment measures.

Current studies suggest that hypoxia is the main reason for the pathophysiological change in high-altitude situations [6,7]. However, under the same hypoxic conditions, some high-altitude residents will remain in good condition, while others may have AAI [8]. Several genes, including ACE, EDN1, ACYP2, RTEL1, and VEGF, are related to hypoxia [9–11]. In hypoxic environments, transcription of various genes, such as endothelial PAS domain-containing protein 1 (EPAS1) and prolyl hydroxylase domain-containing protein 2 (PHD2), is initiated by hypoxia-related pathways [12]. Also, genetic susceptibility has been reported as one of the major determinants of HAPE and AMS. The oxidative stress-related genes CYBA and GSTP1, contributing to endothelial damage under hypobaric hypoxia, are potential candidate genes for HAPE [13–17]. However, there are a few genetic studies on HAR.

MicroRNAs (miRNAs) can inhibit mRNA translation or induce degradation of target mRNAs by binding to complementary sequences in target regions [18,19]. Huang et al. revealed that saliva miR-134-3p and miR-15b-5p could be used as noninvasive biomarkers to predict AMS individuals exposed to high altitude in advance [19]. In a hypoxic atmosphere, the expression levels of miR-16, 20b, 22, 206, and 17/92 are reduced, which can inhibit ion channels, increase pulmonary artery pressure, and cause vascular dysfunction and the loss of cell integrity, promoting the occurrence of HAPE, and miR-155, 23b, 26a can inhibit TGF signals and promote pulmonary pressure, miR-210 can inhibit mitochondrial function. These may promote the occurrence of HAPE [20]. However, the differential expression of miRNAs has not been reported in HAR.

In the present study, we aimed to explore HAR-related miRNAs and mRNAs via analyzing the GSE90500 and GSE75665 datasets from the Gene Expression Omnibus (GEO). Gene Ontology (GO) functional annotations, Kyoto Encyclopedia of Genes and Genomes (KEGG) pathway analysis, potential target gene analysis, and construction of potential miRNA–mRNA network were also conducted. Besides, the HAR cell model was established to validate the results of the bioinformatics analysis via quantitative RT-PCR (qRT-PCR). The present study may help elucidate the pathogenic mechanism and identify valuable diagnostic biomarkers for HAR.

## Materials and methods

### Data source

**Figure 1:**
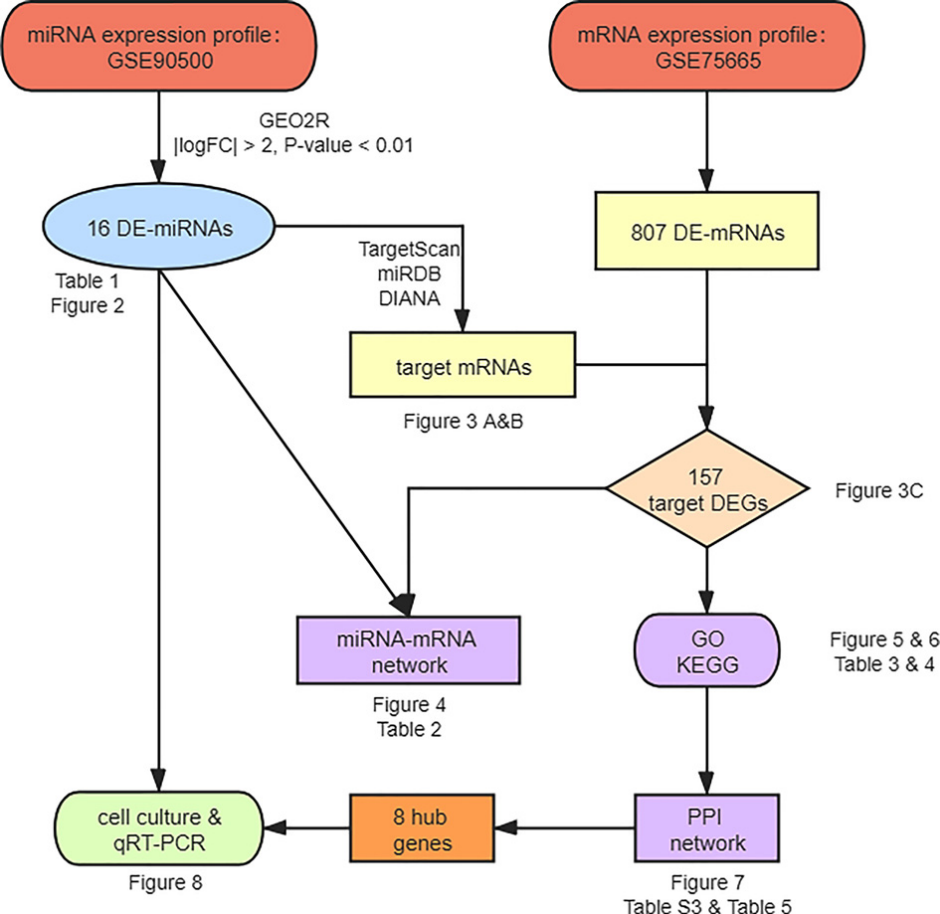
The workflow of bioinformatics analysis

The miRNA expression profile GSE90500 and the mRNA expression profile GSE75665 were obtained from the GEO database. The RNA-seq data of GSE90500 were based on the platform of GPL18058 (Exiqon miRCURY LNA microRNA array, 7th generation) and contained 13 patients with AMS and 9 non-AMS volunteers. The microarray data of GSE75665 were based on the platform GPL11154 (Illumina HiSeq 2000) and contained 5 patients with AMS and 5 non-AMS volunteers. Figure 1 shows the workflow for the study.

### Identification of the differentially expressed miRNA and differentially expressed mRNA

The GEO2R online tool (https://www.ncbi.nlm.nih.gov/geo/geo2r/) based on the R software ‘LIMMA’ package was used to identify differentially expressed miRNA (DE-miRNA) between AMS and non-AMS blood samples of GSE90500 with thresholds of |logFC| > 2 and *P*-value <0.01. A volcano plot was generated to visualize the significant miRNA expression changes using GraphPad Prism 8.0 (GraphPad, San Diego, CA, USA). For GSE75665, differentially expressed mRNAs (DE-mRNAs) related to AMS were obtained from the study of Liu et al. [21].

### The prediction of target genes of DE-miRNA

We used three prediction tools to predict the target mRNAs of DE-miRNA: TargetScan (http://www.targetscan.org/), miRDB (http://mirdb.org/), and DIANA-microT (http://www.microrna.gr/microT). The genes predicted by at least two programs were chosen as the targets of DE-miRNA. We selected the overlapping genes between targets and DE-mRNAs as target differentially expressed genes (DEGs). A heatmap was performed using the R package ‘Pheatmap’. Then, using Cytoscape software [22], the miRNA–mRNA regulatory network was constructed.

### Functional enrichment analysis of miRNA-target DEG

GO annotation and KEGG pathway enrichment analyses were performed by utilizing the Database for Annotation, Visualization, and Integrated Discovery (DAVID, http://david.abcc.Ncifcrf.gov/) online software [23]. *P*<0.05 was set as the cut-off value. The GO analysis included three categories: biological process (BP), cellular component (CC), and molecular function (MF). Heatmaps of DEGs significantly enriched KEGG pathways was performed by the R package ‘Pheatmap’.

### Construction of the protein–protein interaction network

By putting the target DEGs in the Search Tool for the Retrieval of Interacting Genes (STRING, http://string-db.org/) database [24], the interaction relationships among DEGs at the protein level were screened. Then, the protein–protein interaction (PPI) networks for up- and down-regulated target DEGs were constructed under the criterion of a combined score > 0.4. Cytoscape software was used to visualize the networks. The connectivity degrees were calculated through network statistical methods.

### Establishment of HAR cell model

Human retinal microvascular endothelial cells (HRMECs) were purchased from Cell Systems Corporations (catalog no. ACBRI181; Kirkland, WA, USA) [25]. The HRMECs were cultured in M199 medium supplemented with 20% fetal bovine serum, 3 ng/ml FGF-basic, 10 units/ml heparin, and 1% streptomycin/penicillin at 37°C. To establish the hypoxia model, cell culture was performed in a hypoxia chamber filled with an anaerobic gas mixture of 94% N_2_, 5% CO_2_, and 1% O_2_ for 12 h. Control groups were cultured in 95% air and 5% CO_2_.

### Quantitative real-time PCR

Total RNA was extracted from HRMECs by using TRIzol reagent. RNA was reverse-transcribed into complementary DNA using PrimeScript RT Master Mix or miRNA First-Strand Synthesis kits (TaKaRa, Kumamoto, Japan), following the manufacturer’s protocols. qRT-PCR was performed by TB Green Premix ExTaq (TaKaRa, Kumamoto, Japan). The oligonucleotide primers used for PCR amplification were purchased from BioSune Biotechnology (Shanghai, China) and are listed in Supplementary Table S1. ACTB and U6 served as internal control for gene and miRNA expression in analysis, respectively. The relative expression of the DE-miRNAs and the DE-mRNAs was calculated using the 2^−ΔΔ*C*_t_^ method.

### Statistical analysis

The data in the present study are presented as the mean ± SD. The results were analyzed by the two-tailed Student’s *t* test, and *P*<0.05 was considered to show a statistical difference.

## Results

### Identification of the DE-mRNA and the DE-miRNA

**Figure 2:**
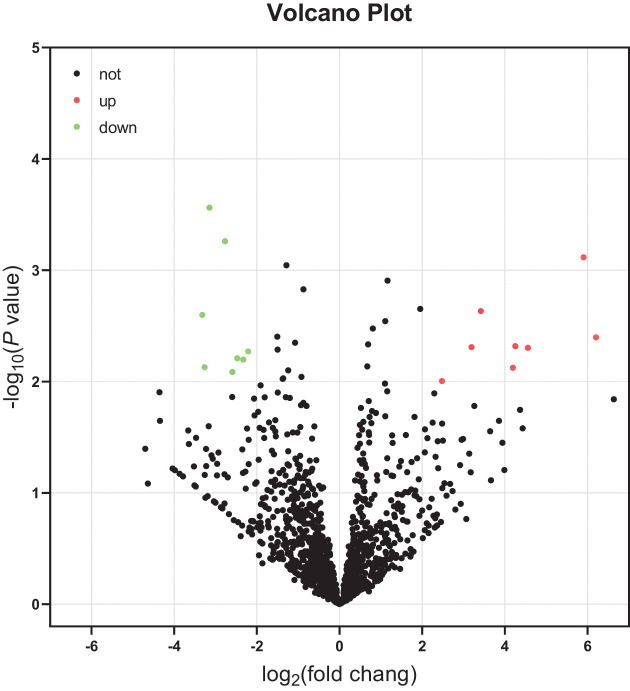
Volcano plot of DE-miRNAs Red dots indicate up-regulated miRNAs and green dots indicate down-regulated miRNAs with *P*<0.01 and |logFC| > 2. Black dots indicate the genes which were not differentially expressed in AMS vs. non-AMS.

According to the analysis of Liu et al. for GSE75665 [21], a total of 807 genes were differentially expressed between the non-AMS and AMS groups, with 271 up-regulated and 535 down-regulated DE-mRNAs (Supplementary Table S2). During an analysis of the GSE90500 dataset, we obtained 16 DE-miRNAs (8 up-regulated and 8 down-regulated) by using the GEO2R online tool (*P*<0.01 and |logFC| > 2) (Table 1). These DE-miRNAs are indicated by a volcano plot (Figure 2). MiR-495 includes miR-495-3p and miR-495-5p, and we discussed them separately in the study, so there were 17 DE-miRNAs (9 up-regulated and 8 down-regulated).

**Table 1:** Up- and down-regulated DE-miRNA

DE-miRNA	LogFC	T	B	*P*-value	Regulation
hsa-miR-3177-3p	−6.20491	−5.3524	−3.024	4.01E-03	Up
hsa-miR-369-3p	−5.90352	−6.64136	−2.0516	7.64E-04	Up
hsa-miR-138-2-3p	−4.5548	−4.49122	−2.6156	4.98E-03	Up
hsa-miR-603	−4.25215	−4.52661	−2.6024	4.81E-03	Up
hsa-miR-495	−4.19332	−3.61489	−2.5279	7.51E-03	Up
hsa-miR-4791	−3.41691	−4.26991	−1.8136	2.33E-03	Up
hsa-miR-424-5p	−3.19308	−4.15642	−2.4021	4.90E-03	Up
hsa-miR-449b-3p	−2.47931	−3.29887	−2.5743	9.89E-03	Up
hsa-miR-23b-5p	3.31787	4.21334	−1.7154	2.53E-03	Down
hsa-miR-1304-3p	3.26306	3.3816	−2.3317	7.43E-03	Down
hsa-miR-1183	3.14353	5.14318	0.0349	2.74E-04	Down
hsa-miR-1255b-5p	2.77088	4.48947	−0.2807	5.49E-04	Down
hsa-miR-15b-5p	2.59052	2.88696	−2.4875	8.20E-03	Down
hsa-miR-1258	2.47358	3.60538	−2.2692	6.18E-03	Down
hsa-miR-3144-3p	2.32697	3.15168	−2.1737	6.34E-03	Down
hsa-miR-155-5p	2.20884	3.06764	−2.1221	5.35E-03	Down

### MiRNA–mRNA network

**Figure 3:**
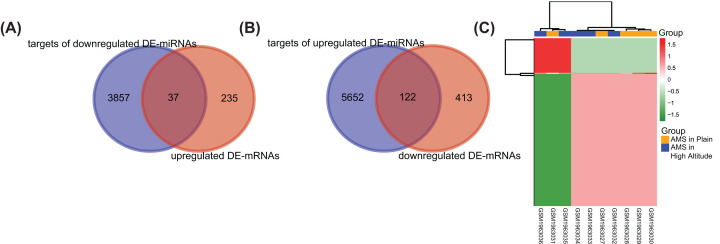
The mRNAs related to AMS (**A**) Overlapped genes between targets of up-regulated DE-miRNAs and down-regulated DE-mRNAs. (**B**) Overlapped genes between targets of down-regulated DE-miRNAs and up-regulated DE-mRNAs. (**C**) Heatmap plot of 157 target DEGs. Green means down-regulation, while red means up-regulation.

**Figure 4:**
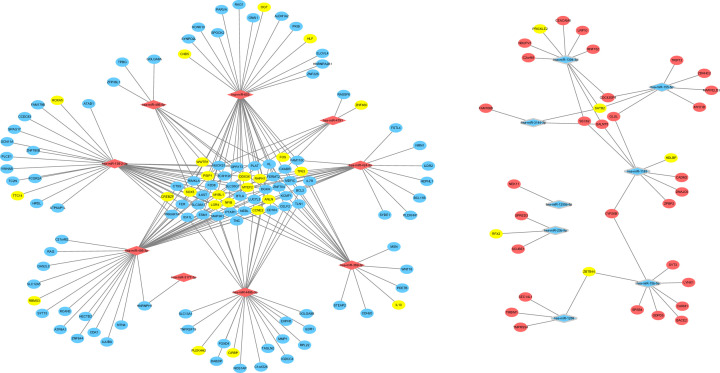
The miRNA–mRNA network in AMS The diamonds stand for DE-miRNAs, and the ellipses stand for target DEGs. The red and blue colors represent up-regulation and down-regulation, respectively. The yellow ellipses represent transcription factors. The lines indicate the regulation relationship between DE-miRNAs and target DEGs.

The target mRNAs of the DE-miRNAs were predicted by the three programs. Besides, by comparing the targets with DE-mRNAs, we only selected the overlapping genes as target DEGs. As shown in Figure 3A and B, 122 out of 5652 predicted targets of up-regulated DE-miRNAs overlapped down-regulated DE-mRNAs, and 37 out of 3857 predicted targets of down-regulated DE-miRNAs overlapped up-regulated DE-mRNAs. After removing 1 duplicate, we got 157 target DEGs, and the expression of these DEGs was visualized in Figure 3C. Then, we constructed the miRNA–mRNA regulatory network (Figure 4), which consisted of 240 miRNA–mRNA pairs in total. Among them, there were 196 up-regulated miRNA–mRNA pairs and 44 down-regulated miRNA–mRNA pairs. Among the target DEGs, there were 29 transcription factors and 128 non-transcription factors. Table 2 listed the target DEGs of DE-miRNAs.

**Table 2:** The target DEGs of DE-miRNA

MiRNA	Up/Down	Count	Target DEGs
hsa-miR-3177-3p	Up	1	*HNRNPH1*
hsa-miR-369-3p	Up	18	*IL10, IL7R, ANLN, CCNE2, CDH20, CELF2, CXADR, KCMF1, KL, MDFIC, MSN, NFIB, PDE7B, PTAR1, STEAP2, TLN1, TP63, WNT16*
hsa-miR-138-2-3p	Up	36	*FCGR2A, DDX3X, ATAD1, ATP6AP1L, BTLA, CCDC83, CREBZF, CTSS, DGKH, FAM179B, FER, GPR173, HOXA5, HPDL, ICA1L, IL6ST, LGR4, MAP3K1, MYBL1, NFIB, NUCKS1, PLAT, PLCE1, PRKAR1A, PSIP1, PTAR1, RIMKLA, SCN11A, SLC35G1, SLC38A1, SPAG17, TC2N, TTC14, YWHAB, ZC3H12C, ZNF780B*
hsa-miR-603	Up	37	*FOS, ALDH1A2, BTLA, CD164, CELF2, CHD5, DGKH, ELOVL4, ESM1, FAM110C, FER, FZD8, GNAI1, GPR173, HLF, HNRNPA2B1, IL6ST, KCMF1, KCNK10, LGR4, LUC7L3, MDFIC, NEBL, NFIB, OGT, PARVA, PKIB, PLAT, PSIP1, RAG1, RAPH1, RIMKLA, SPOCK2, SYNPO2L, TP63, ZNF226, ZNF704*
hsa-miR-495-3P	Up	41	*DDX3X, HNRNPH1, CDK1, AJUBA, ANLN, ATP8A1, C21ORF62, CCNE2, CD164, CELF2, CREBZF, CTSS, DGKH, ESM1, FER, FERMT2, FZD8, GAS2L3, HECTD2, KCMF1, LUC7L3, MAP3K1, MYEF2, NEBL, NFIB, NTN4, NUCKS1, PRKAR1A, PTAR1, RAI2, RAPH1, RBMS3, RCAN3, SLC12A5, SOX5, SYT15, TNC, WWTR1, ZC3H12C, ZNF644, ZNF704*
hsa-miR-495-5p	Up	10	*DDX3X, DGKH, GOLGA8A, IL6ST, KL, SOX5, TPBG, WWTR1, ZC3H12C, ZFP36L1*
hsa-miR-4791	Up	7	*FOS, CXADR, FZD8, NUCKS1, RASSF6, SLC38A1, ZNF460*
hsa-miR-424-5p	Up	19	*IL7R, DDX3X, BCL11B, ANLN, BCL2, BTLA, FAM110C, FERMT2, FSTL4, HEPHL1, ILDR2, MYBL1, MYEF2, NEBL, NRN1, PLEKHH1, SLC35G1, SYDE1, ZNF704*
hsa-miR-449b-3p	Up	27	*BCL2, C1ORF226, CD164, CELF2, CIRBP, DAB2IP, DGKH, EXPH5, FOXD, GOLGA8B, ICA1L, IGDCC4, ILDR1, KL, LGR4, LUC7L3, MAP3K, MMP1, NOS1AP, PLEKHH2, RPL22, SLC13A1, SLC38A1, TAGLN2, TLN1, TNC, TNFRSF19*
hsa-miR-23b-5p	Down	3	*SCUBE1, SPRED3, RFX2*
hsa-miR-1304-3p	Down	10	*C2ORF48, CDC42EP4, GALNT5, LRP10, NDUFV3, PRICKLE2, RNF152, SATB2, CEACAM8, SEC62*
hsa-miR-1183	Down	8	*CADM2, DNAJC6, GALNT5, GLUL, ZPBP2, CYP26B1, HDLB, SEC62*
hsa-miR-1255b-5p	Down	1	*NEK11*
hsa-miR-15b-5p	Down	8	*BACE2, GDPD5, SPSB4, SYT3, ZBTB44, CARM1, CYP26B1, LYNX1*
hsa-miR-1258	Down	4	*SEC14L1, TMBIM1, ZBTB44, TMPRSS4*
hsa-miR-3144-3p	Down	3	*GALNT5, SATB2, FAM166B*
hsa-miR-155-5p	Down	7	*CDC42EP4, GLUL, MARVELD3, SATB2, ZDHHC2, MYO1D, TRIP13*

### Functional annotation analysis of the target DEGs

**Figure 5:**
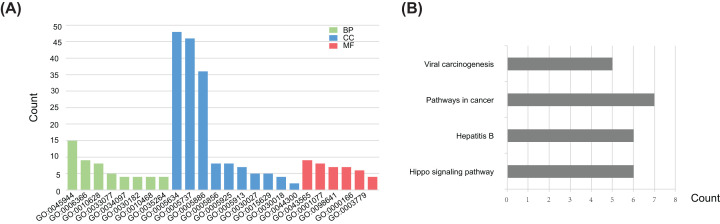
GO enrichment and KEGG pathway analysis of the down-regulated target DEGs (**A**) Significant enriched GO terms of down-regulated target DEGs. Green bars stand for the BP, blue bars stand for the CC, and red bars stand for the MF. (**B**) KEGG pathway analysis of the down-regulated target DEGs.

**Figure 6:**
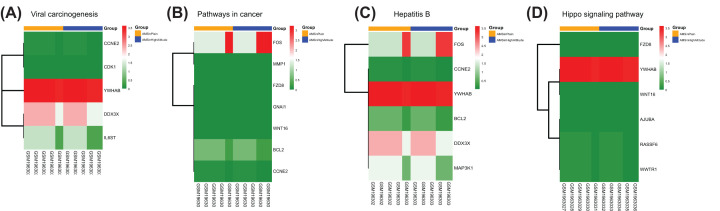
Heatmaps of down-regulated DEGs significantly enriched four KEGG pathways Viral carcinogenesis (**A**), pathways in cancer (**B**), Hepatitis B (**C**), and Hippo signaling pathway (**D**). Green means down-regulation, while red means up-regulation.

To further explore the biological function of the target DEGs, GO enrichment and KEGG pathway analysis were performed using DAVID. GO analysis results showed that acrosome assembly was the only significantly enriched BP for the up-regulated target DEGs. As shown in Figure 5A and Table 3, the down-regulated target DEGs were mainly enriched in the process of RNA expression, such as positive regulation of transcription from RNA polymerase II promoter, positive regulation of gene expression at BP level. On the CC level, the down-regulated DEGs were mainly enriched in the nucleus, cytoplasm, and plasma membrane. For the MF, the down-regulated DEGs were mainly enriched in sequence-specific DNA binding, transcriptional activator activity, and RNA polymerase II core promoter proximal region sequence-specific binding. In addition, KEGG pathway analyses indicated that down-regulated DEGs were significantly enriched in the Hippo signaling pathway (Figure 5B and Table 4), which is associated with the proliferation and migration of vascular endothelial cells [26,27] and can be deactivated by hypoxia [28]. In addition, as shown in Figure 6A–D, heatmaps illustrated the allocation of down-regulated DEGs significantly enriched four KEGG pathways.

**Table 3:** GO enrichment terms of down-regulated target DEGs in AMS

GO ID	GO Term	Category	Count	*P*-value	FDR
GO:0045944	Positive regulation of transcription from RNA polymerase II promoter	BP	15	5.22E-03	7.91E+00
GO:0006366	Transcription from RNA polymerase II promoter	BP	9	2.05E-02	2.78E+01
GO:0010628	Positive regulation of gene expression	BP	8	1.78E-03	2.77E+00
GO:0033077	T-cell differentiation in thymus	BP	5	2.82E-05	4.45E-02
GO:0034097	Response to cytokine	BP	4	4.91E-03	7.47E+00
GO:0030182	Neuron differentiation	BP	4	2.51E-02	3.30E+01
GO:0010468	Regulation of gene expression	BP	4	2.86E-02	3.67E+01
GO:0035264	Multicellular organism growth	BP	4	1.60E-02	2.24E+01
GO:0048041	Focal adhesion assembly	BP	3	1.09E-02	1.58E+01
GO:0048538	Thymus development	BP	3	3.28E-02	4.09E+01
GO:0042100	B-cell proliferation	BP	3	1.89E-02	2.60E+01
GO:1900740	Positive regulation of protein insertion into mitochondrial membrane involved in apoptotic signaling pathway	BP	3	1.67E-02	2.33E+01
GO:0008625	Extrinsic apoptotic signaling pathway via death domain receptors	BP	3	2.61E-02	3.41E+01
GO:0045727	Positive regulation of translation	BP	3	4.80E-02	5.39E+01
GO:0031532	Actin cytoskeleton reorganization	BP	3	3.86E-02	4.62E+01
GO:0034446	Substrate adhesion-dependent cell spreading	BP	3	2.61E-02	3.41E+01
GO:0032835	Glomerulus development	BP	3	1.51E-03	2.36E+00
GO:0072577	Endothelial cell apoptotic process	BP	2	4.54E-02	5.19E+01
GO:0046632	α-β T-cell differentiation	BP	2	3.90E-02	4.66E+01
GO:0005634	Nucleus	CC	48	3.07E-03	3.67E+00
GO:0005737	Cytoplasm	CC	46	4.66E-03	5.52E+00
GO:0005886	Plasma membrane	CC	36	1.88E-02	2.07E+01
GO:0005856	Cytoskeleton	CC	8	7.63E-03	8.90E+00
GO:0005925	Focal adhesion	CC	8	1.00E-02	1.15E+01
GO:0005913	Cell–cell adherens junction	CC	7	1.42E-02	1.60E+01
GO:0030027	Lamellipodium	CC	5	1.66E-02	1.84E+01
GO:0015629	Actin cytoskeleton	CC	5	4.44E-02	4.24E+01
GO:0030018	Z disc	CC	4	3.54E-02	3.56E+01
GO:0044300	Cerebellar mossy fiber	CC	2	3.01E-02	3.11E+01
GO:0043565	Sequence-specific DNA binding	MF	9	1.81E-02	2.14E+01
GO:0001077	Transcriptional activator activity, RNA polymerase II core promoter proximal region sequence-specific binding	MF	8	8.05E-04	1.05E+00
GO:0098641	Cadherin binding involved in cell-cell adhesion	MF	7	1.08E-02	1.34E+01
GO:0000166	Nucleotide binding	MF	7	2.44E-02	2.77E+01
GO:0003779	Actin binding	MF	6	3.31E-02	3.58E+01
GO:0003730	mRNA 3′-UTR binding	MF	4	3.79E-03	4.87E+00

**Table 4:** Enriched KEGG pathway of down-regulated target DEGs associated with AMS

KEGG ID	KEGG Term	Count	FDR	Genes
hsa04390	Hippo signaling pathway	6	2.65E+00	*AJUBA, FZD8, WNT16, RASSF6, YWHAB, WWTR1*
hsa05161	Hepatitis B	6	2.23E+00	*CCNE2, FOS, DDX3X, BCL2, MAP3K1, YWHAB*
hsa05200	Pathways in cancer	7	3.27E+01	*CCNE2, FZD8, FOS, WNT16, GNAI1, BCL2, MMP1*
hsa05203	Viral carcinogenesis	5	3.60E+01	*CCNE2, CDK1, DDX3X, IL6ST, YWHAB*

### PPI network of the target DEGs

**Figure 7:**
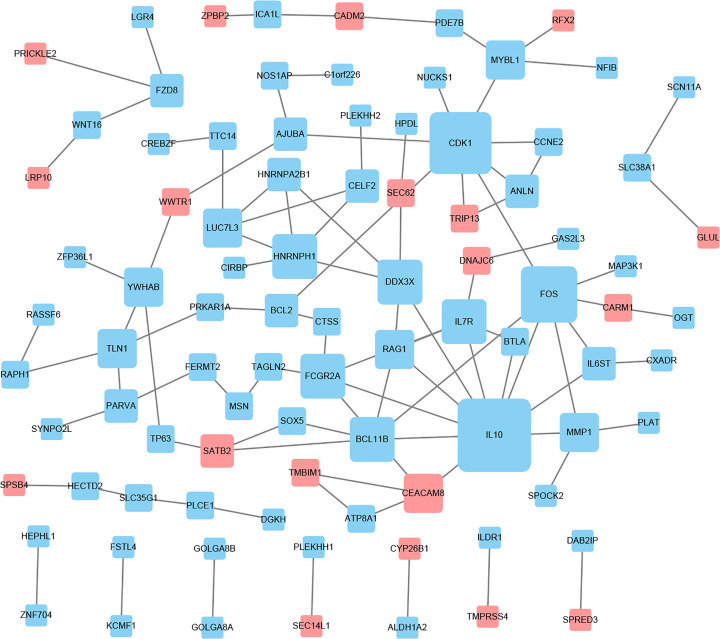
PPI network construction and module analysis The red nodes represent the up-regulated DEGs. The blue nodes represent the down-regulated DEGs. The size of the nodes indicates the degree of the DEGs, and the lines represent the interaction between gene-encoded proteins.

Based on information from the STRING database, the PPI network was constructed and contained 59 nodes and 99 interactions (Figure 7). The details of the PPI network are described in Supplementary Table S3. In this network, with the criteria of filtering degree > 5, eight genes were defined as hub genes, including IL10, CDK1, FOS, HNRNPH1, IL7R, BCL11B, FCGR2A, and DDX3X (Table 5).

**Table 5:** Top eight highest degree nodes

Node	IL10	CDK1	FOS	HNRNPH1	IL7R	BCL11B	FCGR2A	DDX3X
Degree	10	8	7	5	5	5	5	5
Type	Down	Down	Down	Down	Down	Down	Down	Down

### Validation of hub genes in HAR cell model

**Figure 8:**
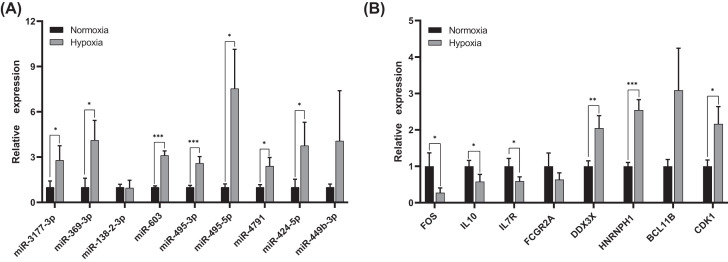
Relative expression levels of DE-miRNAs and hub genes in HRMECs under normoxia and hypoxia conditions by qRT-PCR (**A**) The expression of nine up-regulated miRNAs. (**B**) The expression of eight DEGs. *P<0.05; **P<0.01; ***P<0.001.

To validate the results of bioinformatics analysis, we chose the nine up-regulated DE-miRNAs and eight hub genes for qRT-PCR under normoxia and hypoxia in HRMECs. As shown in Figure 8A and B, miR-3177-3p, miR-369-3p, miR-603, miR-495-3p, miR-495-5p, miR-4791, and miR-424-5p were significantly up-regulated in hypoxia-cultured HRMECs compared with normoxia (*P*<0.05). FOS, IL10, and IL7R were strongly down-regulated under hypoxia conditions (*P*<0.05), which was also consistent with our bioinformatics results. However, the expression of DDX3X, BCL1B, HNRNPH1, and CDK1 was higher than that in normoxia-cultured HRMECs.

## Discussion

HAR is an ocular manifestation of AAI [1], and studies have found a statistically significant correlation between HAR and HACE [3]. Some researchers have proposed that AMS is a mild form of HACE [29,30]. Since the retina and optic nerve act as the directly visible part of the brain, belonging to the central nervous system due to its embryonic origin [31,32], we obtained the DE-miRNAs and DE-mRNAs of HAR by analyzing the AMS datasets and then explored the possible potential target genes of HAR.

MiRNAs can regulate many BPs by negatively regulating the expression of target genes and are thus important targets for studying the genetic susceptibility of diseases [19,33]. Through a series of bioinformatics analyses and experiments, we selected 16 DE-miRNAs and 157 target DEGs related to AMS from GSE90500 and GSE75665 and constructed an miRNA–mRNA network containing 240 relationship pairs. The top eight hub genes—IL7R, FOS, IL10, FCGR2A, DDX3X, CDK1, BCL11B and HNRNPH1—filtered from the PPI network may serve as candidate biomarkers of HAR. The expression of miR-3177-3p, miR-369-3p, miR-603, miR-495-3p, miR-495-5p, miR-4791, miR-424-5p, FOS, IL10, and IL7R were confirmed by qRT-PCR in our HAR cell model to have the same trend as that found in our bioinformatics results. These miRNAs and mRNAs may play an important role in the development of HAR.

FOS, consisting of c-Fos, FosB, Fra1, and Fra2 [34], was significantly down-regulated in our hypoxia-cultured HRMECs. In the lung tissues of hypoxic rats, the expression of c-Fos and FosB was significantly decreased [35], which is consistent with our results. c-Fos promotes the development of inflammatory diseases such as arthritis [36], but it also inhibits inflammation in myeloid and lymphoid cell lineages [37]. In studies on retinal development, it has been found that c-Fos induces inflammatory signals in photoreceptor cells, leading to an increase in VEGF, thus promoting the formation of new blood vessels [38]. Therefore, FOS may be associated with the leakage of the peripheral retinal vessels and retinal hemorrhages in HAR through the regulation of inflammatory constituents. The bioinformatics analysis showed that miR-603 and miR-4791 may down-regulate FOS, and miR-603 can promote the growth of glioma cells via the Wnt/β-catenin pathway [39]. The Wnt pathway mediates many complex biological and pathological processes of retinopathy [40], but there are no reports about miR-603 and this pathway in the development of HAR. Further research about the role of FOS and miR-603 in HAR is needed.

Another inflammation-related gene, IL10, could reduce tissue damage and promote immune tolerance [41]. It was down-regulated in our study, and maybe serve as a biomarker in HAR. Under hypoxic conditions, the decrease in IL10 indicates hypoxia reduces the ability to inhibit immunity and inflammation [42]. However, in the study of Kang et al., mice adapted to the low oxygen environment (10% O_2_) had higher IL10 levels [43]. Therefore, the role of anti-inflammatory markers IL10 in HAR needs further investigation.

IL7R mediates the signaling of IL7, affecting the role of IL7 in regulating the development of T and B cells and the balance of the internal environment of T cells [44], and IL7R is involved in the pathogenesis of several autoimmune diseases, such as rheumatoid arthritis, multiple sclerosis, and systemic lupus erythematosus etc. [45] High altitude also could cause changes in different immune cells [46]. According to our bioinformatics analysis, we found that IL7R was regulated by miR-369-3p and miR-424-5p. It is noted that the induction of miR-369-3p expression inhibits the release of inflammatory cytokines [47]. Furthermore, up-regulated miR-424-5p in hypoxic vascular endothelial cells was shown to stabilize hypoxia-inducible factor-1α (HIF-1α) and fine-tune VEGF, promoting angiogenesis and preserving the integrity of the endothelial barrier [48]. Although the down-regulated IL7R and the up-regulated miR-369-3p and miR-424-5p may play an important role in the progression of HAR, the specific mechanism is not clear and remains to be clarified.

High-altitude retinal hemorrhages and vascular leakage may be caused by the retinal microcirculation disorder under hypoxia conditions at high altitude, but the specific mechanism is not clear. The expression of DDX3X was found to be correlated with overexpression of HIF-1α in breast cancer, indicating the oncogenic role of DDX3X [49]. CDK1 has been shown to promote tumor angiogenesis by stabilizing HIF-1α, and its disruption could inhibit retinal angiogenesis by inducing cell cycle arrest and apoptosis [50]. However, inhibition of miR-495 improves angiogenesis and blood flow recovery after ischemia [51]. Although the expression of DDX3X and CDK1 did not down-regulate in HRMECs as predicted, these genes had high degrees in the PPI network, indicating their potential roles in the development of HAR. It worth mentioning that very little was reported on miR-3177-3p, so the specific function of miR-3177-3p needs further research.

To our knowledge, our study is the first to explore the DE-miRNAs and DEGs of HAR and establish the miRNA–mRNA network through comprehensive bioinformatics analyses. However, there are still some limitations in the present study. First, the data of the GSE90500 and GSE75665 are collected from blood samples, which may be the reason why the expression of several hub genes in HRMECs was inconsistent with the bioinformatics analyses. Then, there are some differences between the analytical methods of GSE90500 and GSE75665, which reduces the reliability of this study to a certain extent. Third, the miRNA–mRNA relationships in HAR were based on target prediction and statistical evidence, which need experimental investigations. Thus, Further studies are required to verify the specific functions of the identified DE-miRNAs and hub genes in HAR.

In conclusion, the present study identified FOS, IL10, IL7R and seven miRNAs as candidate biomarkers of HAR, which may participate in the development of HAR through possible pathways. It has been proposed that HAR is related to individual sensitivity, promoting the effective prevention, and treatment of HAR in the future. Nevertheless, we carried out the systematic and comprehensive bioinformatics analyses to identify the new miRNA and genes of HAR and performed experimental validation in the HAR cell model, which is helpful for understanding the gene changes in HAR and provides novel candidate biomarkers for the diagnosis of HAR.

## Supplementary Material

Supplementary Tables S1-S3

## Data Availability

All data associated with the present paper are already included in the manuscript.

## Abbreviations

AAI: acute high-altitude illness
AMS: acute mountain sickness
BP: biological process
CC: cellular component
DAVID: Database for Annotation, Visualization, and Integrated Discovery
DE-miRNA: differentially expressed miRNA
DE-mRNA: differentially expressed mRNA
DEG: differentially expressed gene
GEO: Gene Expression Omnibus
GO: Gene Ontology
HACE: high-altitude cerebral edema
HAPE: high-altitude pulmonary edema
HAR: high-altitude retinopathy
HIF-1α: hypoxia-inducible factor-1α
HRMEC: human retinal microvascular endothelial cell
KEGG: Kyoto Encyclopedia of Genes and Genomes
MF: molecular function
miRNA: microRNA
qRT-PCR: uantitative RT-PCR
STRING: Search Tool for the Retrieval of Interacting Genes

## References

[1] Houston C.S. (1976) High altitude illness. Disease with protean manifestations. JAMA 236, 2193–2195 10.1001/jama.1976.03270200031025 989810

[2] Seth R.K. and Adelman R.A. (2010) High-altitude retinopathy and optical coherence tomography findings. Semin. Ophthalmol. 25, 13–15 10.3109/08820538.2010.481560 20507191

[3] Wiedman M. and Tabin G.C. (1999) High-altitude retinopathy and altitude illness. Ophthalmology 106, 1924–1926, discussion 1927 10.1016/S0161-6420(99)90402-5 10519586

[4] Tian X. et al. (2018) Retinal changes following rapid ascent to a high-altitude environment. Eye (Lond.) 32, 370–374 10.1038/eye.2017.195 28912514 PMC5811714

[5] Honigman B. et al. (2001) High altitude retinal hemorrhages in a Colorado skier. High Alt. Med. Biol. 2, 539–544 10.1089/152702901753397117 11809095

[6] McFadden D.M. et al. (1981) High-altitude retinopathy. JAMA 245, 581–586 10.1001/jama.1981.03310310023016 7452886

[7] Li K. et al. (2017) Transcriptome reveals the overexpression of a kallikrein gene cluster (KLK1/3/7/8/12) in the Tibetans with high altitude-associated polycythemia. Int. J. Mol. Med. 39, 287–296 10.3892/ijmm.2016.2830 28000848 PMC5358693

[8] MacInnis M.J. and Koehle M.S. (2016) Evidence for and against genetic predispositions to acute and chronic altitude illnesses. High Alt. Med. Biol. 17, 281–293 10.1089/ham.2016.0024 27500591

[9] Jin T. et al. (2019) Association between the IL1R2 rs2072472 polymorphism and high-altitude pulmonary edema risk. Mol. Genet. Genom. Med. 7, e542 10.1002/mgg3.542 30672138 PMC6418374

[10] Hanaoka M. et al. (2009) Polymorphisms of human vascular endothelial growth factor gene in high-altitude pulmonary oedema susceptible subjects. Respirology 14, 46–52 10.1111/j.1440-1843.2008.01420.x 19144048

[11] Rong H. et al. (2017) Association between regulator of telomere elongation helicase1 (RTEL1) gene and HAPE risk: a case-control study. Medicine (Baltimore) 96, e8222 10.1097/MD.0000000000008222 28953687 PMC5626330

[12] Pan H. et al. (2018) Mutations in EPAS1 in congenital heart disease in Tibetans. Biosci. Rep. 38, 6 10.1042/BSR20181389. PMC6435565 30487161

[13] He Y. et al. (2016) Telomere length-related gene ACYP2 polymorphism is associated with the risk of HAPE in Chinese Han population. J. Gene Med. 18, 244–249 10.1002/jgm.2896 27552709

[14] Mishra A. et al. (2012) CYBA and GSTP1 variants associate with oxidative stress under hypobaric hypoxia as observed in high-altitude pulmonary oedema. Clin. Sci. (Lond.) 122, 299–309 10.1042/CS20110205 21973220

[15] Richalet J.P. et al. (2012) Physiological risk factors for severe high-altitude illness: a prospective cohort study. Am. J. Respir. Crit. Care Med. 185, 192–198 10.1164/rccm.201108-1396OC 22071330

[16] Schneider M. et al. (2002) Acute mountain sickness: influence of susceptibility, preexposure, and ascent rate. Med. Sci. Sports Exerc. 34, 1886–1891 10.1097/00005768-200212000-00005 12471292

[17] Bloch K.E. et al. (2009) Effect of ascent protocol on acute mountain sickness and success at Muztagh Ata, 7546 m. High Alt. Med. Biol. 10, 25–32 10.1089/ham.2008.1043 19326598

[18] Lou W. et al. (2018) MicroRNA regulation of liver cancer stem cells{Lou, 2018 #42}. Am J. Cancer Res. 8, 1126–1141 30094089 PMC6079154

[19] Nunez-Iglesias J. et al. (2010) Joint genome-wide profiling of miRNA and mRNA expression in Alzheimer’s disease cortex reveals altered miRNA regulation. PLoS ONE 5, e8898 10.1371/journal.pone.0008898 20126538 PMC2813862

[20] Alam P., Saini N. and Pasha M.A. (2015) MicroRNAs: an apparent switch for high-altitude pulmonary edema. MicroRNA 4, 158–167 10.2174/2211536604666151103121633 26527285

[21] Liu B. et al. (2017) IL-10 dysregulation in acute mountain sickness revealed by transcriptome analysis. Front. Immunol. 8, 628 10.3389/fimmu.2017.00628 28611780 PMC5447681

[22] Smoot M.E. et al. (2011) Cytoscape 2.8: new features for data integration and network visualization. Bioinformatics 27, 431–432 10.1093/bioinformatics/btq675 21149340 PMC3031041

[23] Huang da W., Sherman B.T. and Lempicki R.A. (2009) Systematic and integrative analysis of large gene lists using DAVID bioinformatics resources. Nat. Protoc. 4, 44–57 10.1038/nprot.2008.211 19131956

[24] Szklarczyk D. et al. (2011) The STRING database in 2011: functional interaction networks of proteins, globally integrated and scored. Nucleic Acids Res. 39, D561–D568 10.1093/nar/gkq973 21045058 PMC3013807

[25] Gu C. et al. (2019) miR-590-3p inhibits pyroptosis in diabetic retinopathy by targeting NLRP1 and inactivating the NOX4 signaling pathway. Invest. Ophthalmol. Vis. Sci. 60, 4215–4223 10.1167/iovs.19-27825 31618425

[26] Moon K.H. and Kim J.W. (2018) Hippo signaling circuit and divergent tissue growth in mammalian eye. Mol. Cells 41, 257–263 29665674 10.14348/molcells.2018.0091 PMC5935098

[27] Kim J. et al. (2017) YAP/TAZ regulates sprouting angiogenesis and vascular barrier maturation. J. Clin. Invest. 127, 3441–3461 10.1172/JCI93825 28805663 PMC5669570

[28] Ma B. et al. (2015) Hypoxia regulates Hippo signalling through the SIAH2 ubiquitin E3 ligase. Nat. Cell Biol. 17, 95–103 10.1038/ncb3073 25438054

[29] Bartsch P. et al. (2004) Acute mountain sickness: controversies and advances. High Alt. Med. Biol. 5, 110–124 10.1089/1527029041352108 15265333

[30] Hackett P.H. and Roach R.C. (2001) High-altitude illness. N. Engl. J. Med. 345, 107–114 10.1056/NEJM200107123450206 11450659

[31] Hughes S., Yang H. and Chan-Ling T. (2000) Vascularization of the human fetal retina: roles of vasculogenesis and angiogenesis. Invest. Ophthalmol. Vis. Sci. 41, 1217–1228 10752963

[32] Patton N. et al. (2005) Retinal vascular image analysis as a potential screening tool for cerebrovascular disease: a rationale based on homology between cerebral and retinal microvasculatures. J. Anat. 206, 319–348 10.1111/j.1469-7580.2005.00395.x 15817102 PMC1571489

[33] Beermann J. et al. (2016) Non-coding RNAs in development and disease: background, mechanisms, and therapeutic approaches. Physiol. Rev. 96, 1297–1325 10.1152/physrev.00041.2015 27535639

[34] Tkach V. et al. (2003) Role of the Fos family members, c-Fos, Fra-1 and Fra-2, in the regulation of cell motility. Oncogene 22, 5045–5054 10.1038/sj.onc.1206570 12902987

[35] Singh M. et al. (2018) The MAPK-activator protein-1 signaling regulates changes in lung tissue of rat exposed to hypobaric hypoxia. J. Cell. Physiol. 233, 6851–6865 10.1002/jcp.26556 29665093

[36] Shiozawa S. and Tsumiyama K. (2009) Pathogenesis of rheumatoid arthritis and c-Fos/AP-1. Cell Cycle 8, 1539–1543 10.4161/cc.8.10.8411 19395871

[37] Ray N. et al. (2006) c-Fos suppresses systemic inflammatory response to endotoxin. Int. Immunol. 18, 671–677 10.1093/intimm/dxl004 16569682

[38] Sun Y. et al. (2017) Inflammatory signals from photoreceptor modulate pathological retinal angiogenesis via c-Fos. J. Exp. Med. 214, 1753–1767 10.1084/jem.20161645 28465464 PMC5461000

[39] Guo M. et al. (2015) miR-603 promotes glioma cell growth via Wnt/beta-catenin pathway by inhibiting WIF1 and CTNNBIP1. Cancer Lett. 360, 76–86 10.1016/j.canlet.2015.02.003 25681036

[40] Huang H.M. et al. (2015) Activating the Wnt/beta-catenin pathway did not protect immature retina from hypoxic-ischemic injury. Invest. Ophthalmol. Vis. Sci. 56, 4300–4308 10.1167/iovs.14-16176 26176867

[41] Hilgenberg E. et al. (2014) Interleukin-10-producing B cells and the regulation of immunity. Curr. Top. Microbiol. Immunol. 380, 69–92 10.1007/978-3-662-43492-5_4 25004814

[42] Yuhai G.U. and Zhen Z. (2015) Significance of the changes occurring in the levels of interleukins, SOD and MDA in rat pulmonary tissue following exposure to different altitudes and exposure times. Exp. Ther. Med. 10, 915–920 10.3892/etm.2015.2604 26622414 PMC4533160

[43] Kang J.G. et al. (2016) Low ambient oxygen prevents atherosclerosis. J. Mol. Med. (Berl.) 94, 277–286 10.1007/s00109-016-1386-3 26830628 PMC4803615

[44] Mai H.L. et al. (2014) IL-7 receptor blockade following T cell depletion promotes long-term allograft survival. J. Clin. Invest. 124, 1723–1733 10.1172/JCI66287 24569454 PMC3973119

[45] Wang X.S. et al. (2013) Perspectives of the relationship between IL-7 and autoimmune diseases. Clin. Rheumatol. 32, 1703–1709 10.1007/s10067-013-2360-x 23934388

[46] Khanna K. et al. (2018) High-altitude-induced alterations in gut-immune axis: a review. Int. Rev. Immunol. 37, 119–126 10.1080/08830185.2017.1407763 29231767

[47] Galleggiante V. et al. (2019) Quercetin-induced miR-369-3p suppresses chronic inflammatory response targeting C/EBP-beta. Mol. Nutr. Food Res. 63, e1801390 10.1002/mnfr.201801390 31338984

[48] Ghosh G. et al. (2010) Hypoxia-induced microRNA-424 expression in human endothelial cells regulates HIF-alpha isoforms and promotes angiogenesis. J. Clin. Invest. 120, 4141–4154 10.1172/JCI42980 20972335 PMC2964978

[49] Bol G.M. et al. (2013) Expression of the RNA helicase DDX3 and the hypoxia response in breast cancer. PLoS ONE 8, e63548 10.1371/journal.pone.0063548 23696831 PMC3656050

[50] Gao X. et al. (2019) Cyclin-dependent kinase 1 disruption inhibits angiogenesis by inducing cell cycle arrest and apoptosis. Exp. Ther. Med. 18, 3062–3070 10.3892/etm.2019.7883 31555388 PMC6755431

[51] Welten S.M. et al. (2014) Inhibition of 14q32 microRNAs miR-329, miR-487b, miR-494, and miR-495 increases neovascularization and blood flow recovery after ischemia. Circ. Res. 115, 696–708 10.1161/CIRCRESAHA.114.304747 25085941

