# Supplementary material for: Integrative analysis of miRNA–mRNA network in high altitude retinopathy by bioinformatics analysis
Source: Biosci Rep. 2021 Jan 14;41(1):BSR20200776. doi: 10.1042/BSR20200776 (PMC7809558; doi:10.1042/BSR20200776)
Supplement: Supplementary Tables S1-S3 [file BSR-2020-0776_supp.zip › BSR-2020-0776_suppST1.pdf]

Table S1. The primers of miRNAs and hub genes

Genes	Sequence of primers
hsa-miR-3177-3p	Forward: 5’ CACGGCACTGGGGAC 3’
hsa-miR-369-3p	Forward: 5’ GCGAATAATACATGGTTGAT 3’
hsa-miR-138-2-3p	Forward: 5’ GCTATTTCACGACACCAGG 3’
hsa-miR-603	Forward: 5’ GGCACACACTGCAATTAC 3’
hsa-miR-495-3p	Forward: 5’ AAACAAACATGGTGCACT 3’
hsa-miR-495-5p	Forward: 5’ GCGAAGTTGCCCATGT 3’
hsa-miR-4791	Forward: 5’ GCGTGGATATGATGACTG 3’
hsa-miR-424-5p	Forward: 5’ CAGCAGCAATTCATGTTT 3’
hsa-miR-449b-3p	Forward: 5’ CAGCCACAACTACCCTG 3’
IL10	Forward: 5’ GTTTTCCCTGACCTCCCTCTA 3’
	Reverse: 5’ GCTCCCTGGTTTCTCTTCCTA 3’
CDK1	Forward: 5’ CAGTCTTCAGGATGTGCTTATG 3’
	Reverse: 5’ TACTGACCAGGAGGGATAGAAT 3’
FOS	Forward: 5’ AAGCGGAGACAGACCAACTA 3’
	Reverse: 5’ GCTGCCAGGATGAACTCTA 3’
HNRNPH1	Forward: 5’ GGGTGTTGAAGCATACTGGT 3’
	Reverse: 5’ CAAAGGGAAGTCCTCTAAGC 3’
IL7R	Forward: 5’ CCACTCTTCCTGAGTTCAGTG 3’
	Reverse: 5’ CACCTTAAACCTTGTGACCAA 3’
BCL11B	Forward: 5’ TTGGATTTCAGGAAGGAGACT 3’
	Reverse: 5’ TAGGTTTCAGAGAGCAAAGCA 3’
FCGR2A	Forward: 5’ GATCCCTACTGCTGGTTTCTG 3’
	Reverse: 5’ TCTGGTAATTGGGCTCTTTGT 3’
DDX3X	Forward: 5’ GCCGATTCACATTACTCTGTT 3’
	Reverse: 5’ TTGTTACTCTTCTGGAGCCAT 3’
