# Supplementary material for: Integrative analysis of miRNA–mRNA network in high altitude retinopathy by bioinformatics analysis
Source: Biosci Rep. 2021 Jan 14;41(1):BSR20200776. doi: 10.1042/BSR20200776 (PMC7809558; doi:10.1042/BSR20200776)
Supplement: Supplementary Tables S1-S3 [file BSR-2020-0776_supp.zip › BSR-2020-0776_suppST2.pdf]

Table S2. All differentially expressed mRNAs in AMS.

Accession	Symbol	Description	Chromosome	AG value*	AD value*	Up/Down
NM_003786	ABCC3	ATP-binding cassette, sub-family C (CFTR/MRP), member 3	chr17	-0.271082	0.0189037	Up
NM_004827	ABCG2	ATP-binding cassette, sub-family G (WHITE), member 2	chr4	-2.80436	1.99557	Up
NM_032548	ABTB1	ankyrin repeat and BTB (POZ) domain containing 1	chr3	2.5435	0.159315	Up
NM_172027	ABTB1	ankyrin repeat and BTB (POZ) domain containing 1	chr3	2.54098	0.158104	Up
NR_033429	ABTB1	ankyrin repeat and BTB (POZ) domain containing 1	chr3	2.53987	0.157807	Up
NM_000665	ACHE	acetylcholinesterase (Yt blood group)	chr7	-2.43263	2.10713	Up
NM_181866	ACOT7	acyl-CoA thioesterase 7	chr1	-1.75584	0.125338	Up
NM_181864	ACOT7	acyl-CoA thioesterase 7	chr1	-1.7569	0.127463	Up
NM_181865	ACOT7	acyl-CoA thioesterase 7	chr1	-1.7569	0.127463	Up
NM_030924	ACSBG2	acyl-CoA synthetase bubblegum family member 2	chr19	-4.31914	2.65068	Up
NM_001145320	ADAMTSL2	similar to ADAMTS-like 2; ADAMTS-like 2	chr9	-4.01063	2.58846	Up
NM_014694	ADAMTSL2	similar to ADAMTS-like 2; ADAMTS-like 2	chr9	-4.01063	2.58846	Up
NM_176801	ADD1	adducin 1 (alpha)	chr4	2.15348	0.0768553	Up
NM_014189	ADD1	adducin 1 (alpha)	chr4	2.1532	0.0768757	Up
NM_014190	ADD1	adducin 1 (alpha)	chr4	2.15291	0.0769217	Up
NM_001119	ADD1	adducin 1 (alpha)	chr4	2.15263	0.0769418	Up
NM_199162	ADPRHL1	ADP-ribosylhydrolase like 1	chr13	-4.81427	0.201935	Up
NM_001185095	AIF1L	allograft inflammatory factor 1-like	chr9	-4.16121	2.72727	Up
NM_001185096	AIF1L	allograft inflammatory factor 1-like	chr9	-4.16121	2.72727	Up
NM_031426	AIF1L	allograft inflammatory factor 1-like	chr9	-4.16121	2.72727	Up
NR_033701	AIF1L	allograft inflammatory factor 1-like	chr9	-4.16121	2.72727	Up
NM_004304	ALK	anaplastic lymphoma receptor tyrosine kinase	chr2	-5.35688	0.152331	Up
NR_003291	ANKRD18DP	hypothetical LOC348840	chr3	-3.51528	2.19953	Up
NR_003366	ANKRD20A8P	ankyrin repeat domain 20 family, member A2 pseudogene; ankyrin repeat domain 20B; ankyrin repeat domain 20 family, member A4	chr2	-4.62695	1.99811	Up
NR_002773	AOC4	AOC3 pseudogene	chr17	-4.7643	0.0389177	Up
NM_032487	ARPM1	actin related protein M1	chr3	-3.45236	2.67819	Up
NM_012105	BACE2	beta-site APP-cleaving enzyme 2	chr21	-1.55904	0.0465098	Up
NM_138992	BACE2	beta-site APP-cleaving enzyme 2	chr21	-1.57819	0.0686524	Up
NM_138991	BACE2	beta-site APP-cleaving enzyme 2	chr21	-1.58627	0.0692405	Up
NM_152439	BEST3	bestrophin 3	chr12	-5.01418	0.898932	Up
NM_014299	BRD4	bromodomain containing 4	chr19	1.7686	0.161572	Up
NM_001109662	C12orf51	chromosome 12 open reading frame 51	chr12	0.519481	0.124412	Up
NM_175895	C12orf61	chromosome 12 open reading frame 61	chr12	-5.17275	0.0755593	Up
NM_152914	C17orf103	chromosome 17 open reading frame 103	chr17	1.38853	0.228839	Up
NM_001010979	C1orf189	chromosome 1 open reading frame 189	chr1	-4.29112	0.0615004	Up
NM_182626	C2orf48	chromosome 2 open reading frame 48	chr2	-4.32048	2.85104	Up
NM_207362	C2orf55	chromosome 2 open reading frame 55	chr2	-2.77967	0.106215	Up
NM_001010907	C9orf153	chromosome 9 open reading frame 153	chr9	-2.5482	2.73938	Up
NM_199001	C9orf169	chromosome 9 open reading frame 169	chr9	-3.2339	1.73179	Up
NM_001167675	CADM2	cell adhesion molecule 2	chr3	-2.10085	2.51374	Up
NM_199141	CARM1	coactivator-associated arginine methyltransferase 1	chr19	1.30907	0.330973	Up
NM_007359	CASC3	cancer susceptibility candidate 3	chr17	1.71794	0.0322781	Up
NM_001752	CAT	catalase	chr11	1.13606	0.00320851	Up
NM_138442	CCDC124	coiled-coil domain containing 124	chr19	0.363653	0.101958	Up
NM_001136203	CCDC124	coiled-coil domain containing 124	chr19	0.363164	0.101563	Up
NM_152492	CCDC27	coiled-coil domain containing 27	chr1	-2.64049	3.33747	Up
NM_005623	CCL8	chemokine (C-C motif) ligand 8	chr17	-2.25086	2.14471	Up
NM_015717	CD207	CD207 molecule, langerin	chr2	-4.06276	2.01762	Up
NM_001252	CD70	CD70 molecule	chr19	-4.68976	0.00525736	Up
NM_012121	CDC42EP4	CDC42 effector protein (Rho GTPase binding) 4	chr17	-1.43309	0.0486122	Up

NM_006319	CDIPT	CDP-diacylglycerol--inositol 3-phosphatidyltransferase (phosphatidylinositol synthase)	chr16	1.30379	0.297653	Up
NM_001816	CEACAM8	carcinoembryonic antigen-related cell adhesion molecule 8	chr19	-1.78414	0.0893129	Up
NM_198207	CERS1	growth differentiation factor 1; LAG1 homolog, ceramide synthase 1	chr19	-2.16917	2.23238	Up
NM_000741	CHRM4	cholinergic receptor, muscarinic 4	chr11	-5.35144	0.460784	Up
NM_022467	CHST8	carbohydrate (N-acetylgalactosamine 4-0) sulfotransferase 8	chr19	-4.75136	0.238716	Up
NM_001130028	CLK3	CDC-like kinase 3	chr15	1.08053	0.192541	Up
NM_003992	CLK3	CDC-like kinase 3	chr15	1.0796	0.193108	Up
NM_030813	CLPB	ClpB caseinolytic peptidase B homolog (E. coli)	chr11	-1.35893	0.139529	Up
NM_001846	COL4A2	collagen, type IV, alpha 2	chr13	-4.25895	2.39517	Up
NM_001863	COX6B1	cytochrome c oxidase subunit Vib polypeptide 1 (ubiquitous)	chr19	1.26174	0.114347	Up
NM_144613	COX6B2	cytochrome c oxidase subunit VIb polypeptide 2 (testis)	chr19	-3.20943	2.66172	Up
NM_000758	CSF2	colony stimulating factor 2 (granulocyte-macrophage)	chr5	-2.88241	3.4078	Up
NM_001143775	CTDNEP1	dullard homolog (Xenopus laevis)	chr17	1.48742	0.2174	Up
NM_001008895	CUL4A	cullin 4A	chr13	0.212638	0.0681693	Up
NM_019885	CYP26B1	cytochrome P450, family 26, subfamily B, polypeptide 1	chr2	-0.548049	0.106701	Up
NR_033811	CYP3A5	cytochrome P450, family 3, subfamily A, polypeptide 5	chr7	-5.3175	0.00104589	Up
NM_000777	CYP3A5	cytochrome P450, family 3, subfamily A, polypeptide 5	chr7	-3.06367	2.21402	Up
NM_001190484	CYP3A5	cytochrome P450, family 3, subfamily A, polypeptide 5	chr7	-3.21381	2.51885	Up
NR_033808	CYP3A5	cytochrome P450, family 3, subfamily A, polypeptide 5	chr7	-3.06367	2.21402	Up
NR_033809	CYP3A5	cytochrome P450, family 3, subfamily A, polypeptide 5	chr7	-3.06367	2.21402	Up
NM_001037160	CYS1	cystin 1	chr2	-2.04718	1.6696	Up
NM_015689	DENND2A	DENN/MADD domain containing 2A	chr7	-2.48939	2.3445	Up
NR_024159	DGCR9	DiGeorge syndrome critical region gene 9	chr22	-4.59356	0.0289005	Up
NM_001934	DLX4	distal-less homeobox 4	chr17	-5.04001	0.0258269	Up
NM_138281	DLX4	distal-less homeobox 4	chr17	-4.97947	0.137396	Up
NM_017833	DNAJC28	DnaJ (Hsp40) homolog, subfamily C, member 28	chr21	-4.52492	1.28133	Up
NM_014787	DNAJC6	DnaJ (Hsp40) homolog, subfamily C, member 6	chr1	-2.89376	2.05272	Up
NM_003585	DOC2B	double C2-like domains, beta	chr17	-3.88011	1.90822	Up
NM_031304	DOHH	deoxyhypusine hydroxylase/monooxygenase	chr19	-1.56837	0.220374	Up
NM_004417	DUSP1	dual specificity phosphatase 1	chr5	2.52691	0.0805201	Up
NM_001037283	EIF3B	eukaryotic translation initiation factor 3, subunit B	chr7	1.00128	0.0795791	Up
NM_003751	EIF3B	eukaryotic translation initiation factor 3, subunit B	chr7	0.998911	0.0801967	Up
NM_001978	EPB49	erythrocyte membrane protein band 4.9 (dematin)	chr8	3.88173	1.01491	Up
NM_001114135	EPB49	erythrocyte membrane protein band 4.9 (dematin)	chr8	3.87117	1.0111	Up
NM_001114137	EPB49	erythrocyte membrane protein band 4.9 (dematin)	chr8	3.87093	1.01126	Up
NM_001114139	EPB49	erythrocyte membrane protein band 4.9 (dematin)	chr8	3.8681	1.01123	Up
NM_001114138	EPB49	erythrocyte membrane protein band 4.9 (dematin)	chr8	3.82783	1.00038	Up
NM_001114136	EPB49	erythrocyte membrane protein band 4.9 (dematin)	chr8	3.81288	0.995511	Up
NM_000129	F13A1	coagulation factor XIII, A1 polypeptide	chr6	1.36836	0.10875	Up
NM_182565	FAM100B	family with sequence similarity 100, member B	chr17	1.74275	0.142672	Up
NM_001006655	FAM149A	family with sequence similarity 149, member A	chr4	-4.21875	2.53842	Up
NM_015398	FAM149A	family with sequence similarity 149, member A	chr4	-4.21875	2.53842	Up
NM_024643	FAM164C	family with sequence similarity 164, member C	chr14	-4.24708	2.78381	Up
NM_001042430	FAM164C	family with sequence similarity 164, member C	chr14	-4.31639	2.64518	Up
NM_001099951	FAM166B	family with sequence similarity 166, member B	chr9	-3.10882	1.75618	Up
NM_182614	FAM70B	family with sequence similarity 70, member B	chr13	-4.82215	0.281047	Up
NM_001113541	FAM75A5	family with sequence similarity 75, member A6; family with sequence similarity 75, member A5; family with sequence similarity 75, member A3	chr9	-1.86819	1.8193	Up
NM_015667	FAM75A7	family with sequence similarity 75, member A7	chr9	-1.86819	1.8193	Up
NM_022039	FBXW4	F-box and WD repeat domain containing 4	chr10	0.294617	0.0550208	Up

NM_144997	FLCN	folliculin	chr17	0.190402	0.247132	Up
NM_002569	FURIN	furin (paired basic amino acid cleaving enzyme)	chr15	2.16505	0.390842	Up
NM_001136197	FZR1	fizzy/cell division cycle 20 related 1 (Drosophila)	chr19	0.546008	0.0598432	Up
NM_007278	GABARAP	GABA(A) receptor-associated protein	chr17	3.00235	0.121733	Up
NM_198321	GALNT10	UDP-N-acetyl-alpha-D-galactosamine:polypeptide N-acetylgalactosaminyltransferase 10 (GalNAc-T10)	chr5	0.579354	0.123863	Up
NM_014568	GALNT5	UDP-N-acetyl-alpha-D-galactosamine:polypeptide N-acetylgalactosaminyltransferase 5 (GalNAc-T5)	chr2	-2.57231	3.34176	Up
NM_003857	GALR2	galanin receptor 2	chr17	-2.85669	1.58642	Up
NM_021996	GBGT1	globoside alpha-1,3-N-acetylgalactosaminyltransferase 1	chr9	-0.259782	0.0791317	Up
NM_030792	GDPD5	glycerophosphodiester phosphodiesterase domain containing 5	chr11	-0.357918	0.00302331	Up
NM_152657	GGN	gametogenetin	chr19	-4.20748	1.47658	Up
NM_022562	GH1	growth hormone 1	chr17	-2.41099	2.58004	Up
NM_002065	GLUL	glutamate-ammonia ligase (glutamine synthetase)	chr1	2.90153	0.0948926	Up
NM_001033044	GLUL	glutamate-ammonia ligase (glutamine synthetase)	chr1	2.89825	0.096971	Up
NM_001033056	GLUL	glutamate-ammonia ligase (glutamine synthetase)	chr1	2.88575	0.105024	Up
NM_005273	GNB2	guanine nucleotide binding protein (G protein), beta polypeptide 2	chr7	2.23258	0.196982	Up
NM_016541	GNG13	guanine nucleotide binding protein (G protein), gamma 13	chr16	-4.21834	2.53858	Up
NM_001170880	GPR137	G protein-coupled receptor 137	chr11	-0.559988	0.12039	Up
NM_020155	GPR137	G protein-coupled receptor 137	chr11	-0.568969	0.116787	Up
NM_007223	GPR176	G protein-coupled receptor 176	chr15	-2.99472	3.56468	Up
NM_004489	GPS2	G protein pathway suppressor 2	chr17	1.21388	0.0885204	Up
NM_024915	GRHL2	grainyhead-like 2 (Drosophila)	chr8	-3.7031	3.29259	Up
NM_000837	GRINA	glutamate receptor, ionotropic, N-methyl D-aspartate-associated protein 1 (glutamate binding)	chr8	2.73311	0.238775	Up
NM_001009184	GRINA	glutamate receptor, ionotropic, N-methyl D-aspartate-associated protein 1 (glutamate binding)	chr8	2.73257	0.238926	Up
NM_001004056	GRK4	G protein-coupled receptor kinase 4	chr4	-5.08712	0.16678	Up
NM_182982	GRK4	G protein-coupled receptor kinase 4	chr4	-5.08712	0.16678	Up
NM_001004057	GRK4	G protein-coupled receptor kinase 4	chr4	-5.12773	0.0855583	Up
NM_002099	GYPA	glycophorin A (MNS blood group)	chr4	-2.36078	2.4065	Up
NM_005330	HBE1	hemoglobin, epsilon 1	chr11	-2.66004	2.96306	Up
NM_006402	HBXIP	hepatitis B virus x interacting protein	chr1	-0.430215	0.026505	Up
NM_005336	HDLBP	high density lipoprotein binding protein	chr2	1.63874	0.0973239	Up
NM_203346	HDLBP	high density lipoprotein binding protein	chr2	1.63387	0.0973168	Up
NM_001243900	HDLBP	high density lipoprotein binding protein	chr2	1.61533	0.0932317	Up
NM_005319	HIST1H1C	histone cluster 1, H1c	chr6	-0.343957	0.114138	Up
NM_003523	HIST1H2BE	histone cluster 1, H2bi; histone cluster 1, H2bg; histone cluster 1, H2be; histone cluster 1, H2bf; histone cluster 1, H2bc	chr6	-4.85501	0.127481	Up
NM_080593	HIST1H2BK	histone cluster 1, H2bk	chr6	1.2001	0.0541431	Up
NM_003519	HIST1H2BL	histone cluster 1, H2bl	chr6	-3.36287	4.02543	Up
NM_006735	HOXA2	homeobox A2	chr7	-4.19718	1.53943	Up
NM_018953	HOXC5	homeobox C5	chr12	-2.88637	2.1638	Up
NM_020386	HRASLS	HRAS-like suppressor	chr3	-3.6296	2.16731	Up
NM_153692	HTRA4	HtrA serine peptidase 4	chr8	-2.72513	3.46553	Up
NR_045414	HTT-AS1	HTT antisense RNA (head to head)	chr4	-3.58161	2.79573	Up
NM_001200029	HYAL3	hyaluronoglucosaminidase 3	chr3	-1.925	0.0910147	Up
NM_002178	IGFBP6	insulin-like growth factor binding protein 6	chr12	-5.00651	0.638385	Up
NM_002188	IL13	interleukin 13	chr5	-2.10788	1.74174	Up
NM_052872	IL17F	interleukin 17F	chr6	-1.69884	2.12369	Up
NM_001031853	INSC	inscuteable homolog (Drosophila)	chr11	-4.84994	1.46354	Up
NM_001042536	INSC	inscuteable homolog (Drosophila)	chr11	-4.84994	1.46354	Up
NM_000419	ITGA2B	integrin, alpha 2b (platelet glycoprotein IIb of IIb/IIIa complex, antigen CD41)	chr17	1.3518	0.153345	Up
NR_002808	ITPK1-AS1	chromosome 14 open reading frame 85	chr14	-3.05913	0.0663623	Up

NM_006694	JTB	jumping translocation breakpoint	chr1	0.902751	0.119392	Up
NM_005354	JUND	jun D proto-oncogene	chr19	1.76095	0.129853	Up
NM_001204798	KCNH2	potassium voltage-gated channel, subfamily H (eag-related), member 2	chr7	-1.27598	1.56615	Up
NM_021012	KCNJ12	similar to hkir2.2x; similar to inward rectifying K+ channel negative regulator Kir2.2v; potassium inwardly-rectifying channel, subfamily J, member 12	chr17	-2.42024	2.48348	Up
NM_001199707	KIAA0895	KIAA0895	chr7	-5.01408	0.183199	Up
NM_015314	KIAA0895	KIAA0895	chr7	-5.01408	0.183199	Up
NM_001009880	KIAA0930	chromosome 22 open reading frame 9	chr22	1.16427	0.146833	Up
NM_015264	KIAA0930	chromosome 22 open reading frame 9	chr22	1.16067	0.148829	Up
NM_022046	KLK14	kallikrein-related peptidase 14	chr19	-4.92049	0.289805	Up
NM_139284	LGI4	leucine-rich repeat LGI family, member 4	chr19	-5.27794	0.330126	Up
NM_002309	LIF	leukemia inhibitory factor (cholinergic differentiation factor)	chr22	-3.0145	2.60256	Up
NM_033514	LIMS3	LIM and senescent cell antigen-like domains 3; LIMS3-LOC440895 read-through	chr2	-3.82514	1.8248	Up
NM_001205288	LIMS3L	LIM and senescent cell antigen-like domains 3-like	chr2	-3.82514	1.8248	Up
NR_038876	LOC100129027	hypothetical protein LOC100129027	chr21	-3.2405	1.85948	Up
NR_034022	LOC100131257	similar to hCG19534	chr7	-5.01172	1.5778	Up
NR_024479	LOC100131726	HCC-related HCC-C11_v3	chr8	-2.56437	5.12874	Up
NR_038849	LOC100505633	long intergenic non-protein coding RNA 1133	chr1	-3.42209	2.06242	Up
NR_040772	LOC100505666	uncharacterized LOC100505666	chr1	-4.71481	1.546	Up
NM_001242737	LOC100506688	uncharacterized LOC100506688	chr5	-3.51405	2.24877	Up
NR_027046	LOC145474	hypothetical protein LOC145474	chr14	-3.72799	0.0378783	Up
NR_015429	LOC283050	hypothetical LOC283050	chr10	-4.58319	1.1657	Up
NR_034148	LOC283143	hypothetical protein LOC283143	chr11	-2.93654	0.0478463	Up
NR_036487	LOC283481	hypothetical protein LOC283481	chr13	-3.02857	2.44967	Up
NR_026846	LOC285074	anaphase promoting complex subunit 1 pseudogene	chr2	-0.361369	0.0292292	Up
NR_033883	LOC339529	hypothetical protein LOC339529	chr1	-1.86797	1.59862	Up
NR_015395	LOC541471	hypothetical LOC541471; non-protein coding RNA 152	chr2	-1.82392	0.192856	Up
NR_015368	LOC643837	hypothetical LOC643837	chr1	-1.49353	0.0326869	Up
NR_037195	LOC646324	hypothetical LOC646324	chr2	-2.98673	3.61644	Up
NR_036652	LOC648809	similar to elongation factor Tu GTP binding domain containing 1	chr15	-4.46893	1.67013	Up
NM_032211	LOXL4	lysyl oxidase-like 4	chr10	-5.03289	1.81267	Up
NM_022896	LPIN3	lipin 3	chr20	-4.57565	2.26359	Up
NM_000237	LPL	lipoprotein lipase	chr8	-4.94127	1.26273	Up
NM_014045	LRP10	low density lipoprotein receptor-related protein 10	chr14	2.77135	0.0139082	Up
NM_005512	LRRC32	leucine rich repeat containing 32	chr11	-1.56698	0.130095	Up
NM_001128922	LRRC32	leucine rich repeat containing 32	chr11	-1.56928	0.125497	Up
NM_021246	LY6G6D	lymphocyte antigen 6 complex, locus G6F; lymphocyte antigen 6 complex, locus G6D	chr6	-4.51784	2.49323	Up
NM_001003693	LY6G6F	lymphocyte antigen 6 complex, locus G6F; lymphocyte antigen 6 complex, locus G6D	chr6	-1.87407	0.0431739	Up
NM_023946	LYNX1	Ly6/neurotoxin 1	chr8	-4.70763	0.0247761	Up
NR_038345	MAGI2-AS3	MAGI2 antisense RNA 3	chr7	-2.41461	2.77132	Up
NM_022818	MAP1LC3B	microtubule-associated protein 1 light chain 3 beta	chr16	1.30772	0.0508874	Up
NM_001017967	MARVELD3	MARVEL domain containing 3	chr16	-4.22979	2.01417	Up
NM_014975	MAST1	microtubule associated serine/threonine kinase 1	chr19	-4.50656	0.381489	Up
NM_005297	MCHR1	melanin-concentrating hormone receptor 1	chr22	-3.16555	2.6359	Up
NM_018518	MCM10	minichromosome maintenance complex component 10	chr10	-4.78339	0.00586942	Up
NM_182751	MCM10	minichromosome maintenance complex component 10	chr10	-4.78339	0.00586942	Up
NR_002211	MEIS3P1	Meis homeobox 3 pseudogene 1	chr17	-3.23718	2.08609	Up
NM_024109	METTL22	chromosome 16 open reading frame 68	chr16	-1.56828	0.00854291	Up
NR_031652	MIR1250	microRNA 1250	chr17	-4.44854	2.70505	Up
NR_029683	MIR142	microRNA 142	chr17	-0.886467	0.226433	Up
NR_031747	MIR2110	microRNA 2110	chr10	-3.5467	3.5578	Up

NR_036162	MIR3194	microRNA 3194	chr20	-1.86797	1.59862	Up
NR_036271	MIR4315-2	microRNA 4315-2	chr17	-3.7217	0.0411351	Up
NR_039616	MIR4420	microRNA 4420	chr1	-5.29772	0.0864646	Up
NR_039743	MIR4518	microRNA 4518	chr16	-4.6678	1.59285	Up
NR_039814	MIR4668	microRNA 4668	chr9	-2.01261	4.02521	Up
NR_039839	MIR4690	microRNA 4690	chr11	-5.09179	0.426417	Up
NR_039865	MIR4715	microRNA 4715	chr15	-2.23623	2.6908	Up
NR_030342	MIR611	microRNA 611	chr11	-5.40944	0.283226	Up
NR_030378	MIR648	microRNA 648	chr22	-2.55887	2.48348	Up
NR_029510	MIR93	microRNA 93	chr7	-5.04601	0.821826	Up
NM_001164105	MOV10L1	Mov10l1, Moloney leukemia virus 10-like 1, homolog (mouse)	chr22	-2.26354	2.79759	Up
NM_001039165	MRGPRE	MAS-related GPR, member E	chr11	-1.75086	0.172669	Up
NM_021019	MYL6	myosin, light chain 6, alkali, smooth muscle and non-muscle	chr12	2.02724	0.0900688	Up
NM_079423	MYL6	myosin, light chain 6, alkali, smooth muscle and non-muscle	chr12	2.01606	0.0979108	Up
NM_181526	MYL9	myosin, light chain 9, regulatory	chr20	0.338936	0.0636001	Up
NM_015194	MYO1D	myosin ID	chr17	-1.66713	0.0390154	Up
NM_001185012	NDUFA2	NADH dehydrogenase (ubiquinone) 1 alpha subcomplex, 2, 8kDa	chr5	-0.222949	0.0150226	Up
NM_002488	NDUFA2	NADH dehydrogenase (ubiquinone) 1 alpha subcomplex, 2, 8kDa	chr5	-0.22563	0.0155149	Up
NR_033697	NDUFA2	NADH dehydrogenase (ubiquinone) 1 alpha subcomplex, 2, 8kDa	chr5	-0.222795	0.0150121	Up
NM_199069	NDUFAF3	NADH dehydrogenase (ubiquinone) 1 alpha subcomplex, assembly factor 3	chr3	0.277097	0.0178913	Up
NM_021075	NDUFV3	NADH dehydrogenase (ubiquinone) flavoprotein 3, 10kDa	chr21	-0.503542	0.0825363	Up
NM_145910	NEK11	NIMA (never in mitosis gene a)- related kinase 11	chr3	-5.12316	0.00341143	Up
NM_176677	NHLRC4	hypothetical protein FLJ36208; chromosome 16 open reading frame 11	chr16	-2.9802	0.0300148	Up
NM_138329	NLRP6	NLR family, pyrin domain containing 6	chr11	-0.315439	0.114635	Up
NM_148896	NPB	neuropeptide B	chr17	-5.01543	0.0234003	Up
NM_001126181	NRGN	neurogranin (protein kinase C substrate, RC3)	chr11	2.84043	0.18809	Up
NM_006176	NRGN	neurogranin (protein kinase C substrate, RC3)	chr11	2.84043	0.18809	Up
NM_001195755	O3FAR1	free fatty acid receptor 4	chr10	-2.87307	2.41672	Up
NM_181745	O3FAR1	G protein-coupled receptor 120	chr10	-2.87307	2.41672	Up
NM_018324	OLAH	oleoyl-ACP hydrolase	chr10	-3.72528	1.8605	Up
NM_001039702	OLAH	oleoyl-ACP hydrolase	chr10	-3.73066	1.87127	Up
NM_001004695	OR2T33	olfactory receptor, family 2, subfamily T, member 33	chr1	-3.25206	2.64352	Up
NM_001134693	OST4	dolichyl-diphosphooligosaccharide--protein glycosyltransferase subunit 4	chr2	1.71918	0.0498223	Up
NM_002563	P2RY1	purinergic receptor P2Y, G-protein coupled, 1	chr3	-4.61963	1.32235	Up
NM_012387	PADI4	peptidyl arginine deiminase, type IV	chr1	1.07708	0.18916	Up
NM_138320	PCSK6	proprotein convertase subtilisin/kexin type 6	chr15	-1.76678	0.000430018	Up
NM_138321	PCSK6	proprotein convertase subtilisin/kexin type 6	chr15	-1.76678	0.000430018	Up
NM_001080471	PEAR1	platelet endothelial aggregation receptor 1	chr1	-1.65595	0.00706407	Up
NM_002630	PGC	progastricsin (pepsinogen C)	chr6	-3.8353	2.7944	Up
NM_018425	PI4K2A	phosphatidylinositol 4-kinase type 2 alpha	chr10	-0.388399	0.0765612	Up
NM_138295	PKD1L1	polycystic kidney disease 1 like 1	chr7	-4.62718	0.726597	Up
NM_001105572	PLA2G2C	phospholipase A2, group IIC	chr1	-2.40583	0.245657	Up
NM_001170944	PNMA6C	similar to Paraneoplastic antigen-like protein 6A	chrX	-2.58376	0.0276232	Up
NM_020709	PNMAL2	PNMA-like 2	chr19	-3.83543	2.38303	Up
NM_024299	PPDPF	pancreatic progenitor cell differentiation and proliferation factor homolog (zebrafish)	chr20	1.64419	0.0682357	Up
NM_014931	PPP6R1	SAPS domain family, member 1	chr19	2.09185	0.0650608	Up
NM_020229	PRDM11	PR domain containing 11	chr11	-4.83524	0.0449153	Up
NM_021619	PRDM12	PR domain containing 12	chr9	-1.96694	1.89931	Up
NM_198859	PRICKLE2	prickle homolog 2 (Drosophila)	chr3	-2.79161	2.21457	Up
NM_000951	PRRG2	proline rich Gla (G-carboxyglutamic acid) 2	chr19	-4.01957	2.27514	Up
NM_002808	PSMD2	proteasome (prosome, macropain) 26S subunit, non-ATPase, 2	chr3	0.817894	0.0248793	Up
NM_198715	PTGER3	prostaglandin E receptor 3 (subtype EP3)	chr1	-2.79589	3.23478	Up

NM_198719	PTGER3	prostaglandin E receptor 3 (subtype EP3)	chr1	-3.47677	2.07734	Up
NM_020387	RAB25	RAB25, member RAS oncogene family	chr1	-2.76419	2.52019	Up
NM_001032726	RAB41	RAB41, member RAS oncogene family	chrX	-5.15343	0.94513	Up
NR_033735	REREP3	arginine-glutamic acid dipeptide (RE) repeats pseudogene 3	chr15	-3.74102	1.76047	Up
NM_000635	RFX2	regulatory factor X, 2 (influences HLA class II expression)	chr19	-0.204769	0.0528769	Up
NM_134433	RFX2	regulatory factor X, 2 (influences HLA class II expression)	chr19	-0.209809	0.0553161	Up
NM_001145664	RFX8	hypothetical protein LOC731220	chr2	-2.30362	2.74298	Up
NM_138616	RHCE	Rh blood group, CcEe antigens	chr1	-1.85231	0.0111513	Up
NM_138617	RHCE	Rh blood group, CcEe antigens	chr1	-1.76834	0.0235469	Up
NM_020639	RIPK4	receptor-interacting serine-threonine kinase 4	chr21	-3.75601	3.62543	Up
NM_173557	RNF152	ring finger protein 152	chr18	-1.99256	3.98512	Up
NM_001193318	RNF212	ring finger protein 212	chr4	-3.12652	2.60709	Up
NM_001207033	RNF40	ring finger protein 40	chr16	1.25047	0.00530318	Up
NM_014771	RNF40	ring finger protein 40	chr16	1.25047	0.00530318	Up
NM_001207034	RNF40	ring finger protein 40	chr16	1.20526	0.00732352	Up
NM_002957	RXRA	retinoid X receptor, alpha	chr9	2.07794	0.0178937	Up
NM_001035	RYR2	ryanodine receptor 2 (cardiac)	chr1	-2.93573	2.3497	Up
NM_005980	S100P	S100 calcium binding protein P	chr4	-1.75169	0.0807778	Up
NM_001172517	SATB2	SATB homeobox 2	chr2	-3.66869	2.21163	Up
NM_015265	SATB2	SATB homeobox 2	chr2	-3.66869	2.21163	Up
NR_003006	SCARNA6	small Cajal body-specific RNA 5; small Cajal body-specific RNA 6	chr2	-4.73664	0.299982	Up
NM_173050	SCUBE1	signal peptide, CUB domain, EGF-like 1	chr22	-4.01962	2.4495	Up
NM_138969	SDR16C5	short chain dehydrogenase/reductase family 16C, member 5	chr8	-2.43022	2.22616	Up
NM_001204408	SEC14L1	SEC14-like 1 (S. cerevisiae); SEC14-like 1 pseudogene	chr17	2.24625	0.0632924	Up
NM_001039573	SEC14L1	SEC14-like 1 (S. cerevisiae); SEC14-like 1 pseudogene	chr17	2.24157	0.0564299	Up
NM_001144001	SEC14L1	SEC14-like 1 (S. cerevisiae); SEC14-like 1 pseudogene	chr17	2.45134	0.140073	Up
NM_003262	SEC62	SEC62 homolog (S. cerevisiae)	chr3	1.15503	0.0546928	Up
NR_038270	SHARPIN	SHANK-associated RH domain interactor	chr8	1.37299	0.0778521	Up
NM_030974	SHARPIN	SHANK-associated RH domain interactor	chr8	1.41702	0.0737667	Up
NM_001128588	SLC14A1	solute carrier family 14 (urea transporter), member 1 (Kidd blood group)	chr18	0.496515	1.67412	Up
NM_001146036	SLC14A1	solute carrier family 14 (urea transporter), member 1 (Kidd blood group)	chr18	0.495388	1.67624	Up
NM_015865	SLC14A1	solute carrier family 14 (urea transporter), member 1 (Kidd blood group)	chr18	0.492064	1.68037	Up
NM_001146037	SLC14A1	solute carrier family 14 (urea transporter), member 1 (Kidd blood group)	chr18	0.47574	1.66796	Up
NM_017875	SLC25A38	solute carrier family 25, member 38	chr3	0.0730697	0.210955	Up
NM_001003841	SLC6A19	solute carrier family 6 (neutral amino acid transporter), member 19	chr5	-2.89546	2.78333	Up
NR_002581	SNORA72	small nucleolar RNA, H/ACA box 72	chr8	-2.33227	2.71111	Up
NR_002433	SNORD12C	small nucleolar RNA, C/D box 12C; small nucleolar RNA, C/D box 12B; small nucleolar RNA, C/D box 12	chr20	-5.28831	0.407631	Up
NR_002564	SNORD26	small nucleolar RNA, C/D box 26	chr11	-4.89873	1.62507	Up
NR_003065	SNORD84	small nucleolar RNA, C/D box 117; small nucleolar RNA, C/D box 84	chr6	-4.72812	1.42938	Up
NM_003118	SPARC	secreted protein, acidic, cysteine-rich (osteonectin)	chr5	1.26185	0.0448477	Up
NM_014471	SPINK4	serine peptidase inhibitor, Kazal type 4	chr9	-4.77126	2.70275	Up
NM_001042522	SPRED3	sprouty-related, EVH1 domain containing 3	chr19	-2.01519	2.39417	Up
NM_080862	SPSB4	splA/ryanodine receptor domain and SOCS box containing 4	chr3	-2.04749	4.09498	Up
NM_003126	SPTA1	spectrin, alpha, erythrocytic 1 (elliptocytosis 2)	chr1	-2.53644	2.11165	Up
NM_013442	STOML2	stomatin (EPB72)-like 2	chr9	-0.296261	0.115115	Up
NM_133625	SYN2	synapsin II	chr3	-3.52326	3.21204	Up
NM_001160328	SYT3	synaptotagmin III	chr19	-1.55513	1.60569	Up
NM_001160329	SYT3	synaptotagmin III	chr19	-1.55513	1.60569	Up
NM_032298	SYT3	synaptotagmin III	chr19	-1.55513	1.60569	Up
NM_001178054	TAC3	tachykinin 3	chr12	-3.2635	4.44798	Up

NM_013251	TAC3	tachykinin 3	chr12	-3.2635	4.44798	Up
NR_033654	TAC3	tachykinin 3	chr12	-3.2635	4.44798	Up
NM_001058	TACR1	tachykinin receptor 1	chr2	-2.28168	1.86305	Up
NM_001057	TACR2	tachykinin receptor 2	chr10	-4.18281	2.28519	Up
NM_024682	TBC1D17	TBC1 domain family, member 17	chr19	0.727149	0.0595625	Up
NM_001168222	TBC1D17	TBC1 domain family, member 17	chr19	0.716533	0.0614423	Up
NM_002536	TBC1D25	TBC1 domain family, member 25	chrX	0.514717	0.114001	Up
NM_001136139	TCF3	transcription factor 3 (E2A immunoglobulin enhancer binding factors E12/E47)	chr19	0.687728	0.0341902	Up
NM_007113	TCHH	trichohyalin	chr1	-2.44955	2.54209	Up
NM_001130011	TEX101	testis expressed 101	chr19	-2.18794	2.78909	Up
NM_031451	TEX101	testis expressed 101	chr19	-2.67838	3.84279	Up
NM_003246	THBS1	thrombospondin 1	chr15	-0.596752	0.136857	Up
NM_012458	TIMM13	translocase of inner mitochondrial membrane 13 homolog (yeast)	chr19	-0.319254	0.00591733	Up
NM_022152	TMBIM1	transmembrane BAX inhibitor motif containing 1	chr2	1.41917	0.170189	Up
NM_001080532	TMC3	transmembrane channel-like 3	chr15	-5.34424	1.28059	Up
NR_024547	TMEM11	transmembrane protein 11	chr17	-0.616775	0.00803816	Up
NM_001136103	TMEM132C	transmembrane protein 132C	chr12	-3.62493	2.20297	Up
NM_001173551	TMPRSS4	transmembrane protease, serine 4	chr11	-2.94836	2.10983	Up
NM_001173552	TMPRSS4	transmembrane protease, serine 4	chr11	-2.94836	2.10983	Up
NM_019894	TMPRSS4	transmembrane protease, serine 4	chr11	-2.94836	2.10983	Up
NM_003286	TOP1	topoisomerase (DNA) I	chr20	1.19683	0.0741287	Up
NM_182752	TPRG1L	tumor protein p63 regulated 1-like	chr1	0.710739	0.0113118	Up
NM_001166260	TRIP13	thyroid hormone receptor interactor 13	chr5	-4.53657	1.28621	Up
NM_001130698	TRPC3	transient receptor potential cation channel, subfamily C, member 3	chr4	-2.1125	0.247612	Up
NM_003305	TRPC3	transient receptor potential cation channel, subfamily C, member 3	chr4	-2.1125	0.247612	Up
NM_004615	TSPAN7	tetraspanin 7	chrX	-2.66244	2.21302	Up
NM_001159726	TSPO2	benzodiazapine receptor (peripheral)-like 1	chr6	-2.91	1.8328	Up
NM_001010873	TSPO2	benzodiazapine receptor (peripheral)-like 1	chr6	-2.91082	1.83115	Up
NR_024506	TTL	twelve-thirteen translocation leukemia gene	chr13	-2.29818	1.9621	Up
NM_021009	UBC	ubiquitin C	chr12	3.73586	0.230802	Up
NM_014501	UBE2S	ubiquitin-conjugating enzyme E2S	chr19	-0.636334	0.0166064	Up
NM_020818	UNC79	KIAA1409	chr14	-2.5829	1.55899	Up
NM_001184831	VSIG4	V-set and immunoglobulin domain containing 4	chrX	-2.80729	0.137268	Up
NM_001100431	VSIG4	V-set and immunoglobulin domain containing 4	chrX	-2.82693	0.161492	Up
NM_001178003	WDR66	WD repeat domain 66	chr12	-3.77858	2.34604	Up
NM_016145	WDR83OS	chromosome 19 open reading frame 56	chr19	0.522786	0.0461492	Up
NM_021197	WFDC1	WAP four-disulfide core domain 1	chr16	-2.88313	3.66032	Up
NM_001168280	WWTR1	WW domain containing transcription regulator 1	chr3	-4.1024	2.47711	Up
NM_015472	WWTR1	WW domain containing transcription regulator 1	chr3	-4.1024	2.47711	Up
NR_001564	XIST	X (inactive)-specific transcript (non-protein coding)	chrX	-1.89094	1.5697	Up
NM_014155	ZBTB44	zinc finger and BTB domain containing 44	chr11	0.286108	0.0613163	Up
NM_016353	ZDHHC2	zinc finger, DHHC-type containing 2	chr8	-0.424285	0.0691713	Up
NM_199451	ZNF365	zinc finger protein 365	chr10	-4.26027	2.64193	Up
NM_198844	ZPBP2	zona pellucida binding protein 2	chr17	-1.66965	1.71971	Up
NM_199321	ZPBP2	zona pellucida binding protein 2	chr17	-1.66965	1.71971	Up
NM_138326	ACMSD	aminocarboxymuconate semialdehyde decarboxylase	chr2	-5.04631	0.28079	Down
NM_002390	ADAM11	ADAM metallopeptidase domain 11	chr17	-3.32933	2.62494	Down
NM_006988	ADAMTS1	ADAM metallopeptidase with thrombospondin type 1 motif, 1	chr21	-3.59555	2.09212	Down
NM_001167749	ADCY10	adenylate cyclase 10 (soluble)	chr1	-3.7521	2.55051	Down
NM_018417	ADCY10	adenylate cyclase 10 (soluble)	chr1	-3.7521	2.55051	Down
NM_001146337	AFAP1L1	actin filament associated protein 1-like 1	chr5	-3.82703	3.23292	Down
NM_152406	AFAP1L1	actin filament associated protein 1-like 1	chr5	-3.82703	3.23292	Down
NM_032876	AJUBA	jub, ajuba homolog (Xenopus laevis)	chr14	-3.99989	3.16282	Down
NM_001206897	ALDH1A2	aldehyde dehydrogenase 1 family, member A2	chr15	-3.0893	3.94845	Down
NM_003888	ALDH1A2	aldehyde dehydrogenase 1 family, member A2	chr15	-3.0893	3.94845	Down

NM_170696	ALDH1A2	aldehyde dehydrogenase 1 family, member A2	chr15	-3.0893	3.94845	Down
NM_000693	ALDH1A3	aldehyde dehydrogenase 1 family, member A3	chr15	-3.36183	2.47341	Down
NM_001182	ALDH7A1	aldehyde dehydrogenase 7 family, member A1	chr5	-2.82277	3.85447	Down
NM_001201377	ALDH7A1	aldehyde dehydrogenase 7 family, member A1	chr5	-2.82277	3.85447	Down
NM_001202404	ALDH7A1	aldehyde dehydrogenase 7 family, member A1	chr5	-2.82277	3.85447	Down
NM_001140	ALOX15	arachidonate 15-lipoxygenase	chr17	-1.83671	1.91292	Down
NM_000478	ALPL	alkaline phosphatase, liver/bone/kidney	chr1	2.26731	0.238428	Down
NM_001127501	ALPL	alkaline phosphatase, liver/bone/kidney	chr1	2.25143	0.239334	Down
NM_001177520	ALPL	alkaline phosphatase, liver/bone/kidney	chr1	2.24503	0.239663	Down
NR_027995	ANKRD20A9P	ankyrin repeat domain 20 family, member A9, pseudogene	chr13	-3.67273	2.94013	Down
NM_018685	ANLN	anillin, actin binding protein	chr7	-4.83879	1.13463	Down
NM_001170	AQP7	aquaporin 7	chr9	-2.09961	2.22407	Down
NR_037901	ARHGEF26-AS	ARHGEF26 antisense RNA 1	chr3	-5.45634	1.05639	Down
NM_001162491	ARL13A	ADP-ribosylation factor-like 13A	chrX	-3.56677	2.98913	Down
NM_001012990	ARL13A	ADP-ribosylation factor-like 13A	chrX	-3.48688	2.82936	Down
NM_005737	ARL4C	ADP-ribosylation factor-like 4C	chr2	1.18375	1.02221	Down
NM_004312	ARR3	arrestin 3, retinal (X-arrestin)	chrX	-4.3776	2.01559	Down
NM_004313	ARRB2	arrestin, beta 2	chr17	2.81341	0.350026	Down
NM_199004	ARRB2	arrestin, beta 2	chr17	2.81121	0.349701	Down
NM_001168530	ASB9	ankyrin repeat and SOCS box-containing 9	chrX	-3.75065	3.13383	Down
NR_033769	ASB9P1	ankyrin repeat and SOCS box containing 9 pseudogene 1	chr15	-2.73399	4.18728	Down
NM_032810	ATAD1	ATPase family, AAA domain containing 1	chr10	-2.03712	1.6583	Down
NM_001017971	ATP6AP1L	ATPase, H+ transporting, lysosomal accessory protein 1-like	chr5	-3.05386	2.29422	Down
NM_003945	ATP6V0E1	ATPase, H+ transporting, lysosomal 9kDa, V0 subunit e1	chr5	1.3812	0.264677	Down
NM_006095	ATP8A1	ATPase, aminophospholipid transporter (APLT), class I, type 8A, member 1	chr4	-1.0581	1.7198	Down
NM_001105529	ATP8A1	ATPase, aminophospholipid transporter (APLT), class I, type 8A, member 1	chr4	-1.05864	1.72017	Down
NM_178537	B4GALNT4	beta-1,4-N-acetyl-galactosaminyl transferase 4	chr11	-3.6251	2.14166	Down
NM_176824	BBS7	Bardet-Biedl syndrome 7	chr4	-3.42083	1.89684	Down
NM_138576	BCL11B	B-cell CLL/lymphoma 11B (zinc finger protein)	chr14	0.0828983	1.34442	Down
NM_022898	BCL11B	B-cell CLL/lymphoma 11B (zinc finger protein)	chr14	0.0761614	1.34486	Down
NM_000633	BCL2	B-cell CLL/lymphoma 2	chr18	-0.779091	1.45807	Down
NM_032735	BEST3	bestrophin 3	chr12	-5.34988	0.065453	Down
NM_001139444	BET3L	BET3 like (S. cerevisiae)	chr6	-1.86541	1.81662	Down
NM_003571	BFSP2	beaded filament structural protein 2, phakinin	chr3	-3.1837	2.26074	Down
NR_002730	BRD7P3	bromodomain containing 7 pseudogene 3	chr6	-2.85449	3.20051	Down
NM_181780	BTLA	B and T lymphocyte associated	chr3	-2.22779	1.80664	Down
NM_001085357	BTLA	B and T lymphocyte associated	chr3	-2.25602	1.80419	Down
NM_001130446	C10orf131	chromosome 10 open reading frame 131	chr10	-4.12162	3.63769	Down
NM_001145024	C11orf34	chromosome 11 open reading frame 34	chr11	-3.67797	2.10534	Down
NM_173525	C11orf42	chromosome 11 open reading frame 42	chr11	-3.1691	4.10804	Down
NM_018169	C12orf35	chromosome 12 open reading frame 35	chr12	1.64587	0.952661	Down
NM_174943	C14orf178	chromosome 14 open reading frame 178	chr14	-5.26712	0.441866	Down
NM_001173978	C14orf178	chromosome 14 open reading frame 178	chr14	-5.31413	0.535884	Down
NR_027123	C14orf33	chromosome 14 open reading frame 33	chr14	-3.80748	2.62752	Down
NM_032413	C15orf48	chromosome 15 open reading frame 48	chr15	-4.8937	1.65238	Down
NM_197955	C15orf48	chromosome 15 open reading frame 48	chr15	-4.8937	1.65238	Down
NM_207445	C15orf54	chromosome 15 open reading frame 54	chr15	-2.92693	3.62371	Down
NM_175741	C15orf55	chromosome 15 open reading frame 55	chr15	-2.25308	2.94828	Down
NM_001039905	C15orf56	chromosome 15 open reading frame 56	chr15	-3.76494	3.79444	Down
NM_001042367	C15orf60	chromosome 15 open reading frame 60	chr15	-3.49321	2.69699	Down
NM_001170754	C1orf127	chromosome 1 open reading frame 127	chr1	-4.01413	1.79034	Down
NM_001085375	C1orf226	chromosome 1 open reading frame 226	chr1	-4.04952	2.59897	Down
NM_181643	C1orf88	chromosome 1 open reading frame 88	chr1	-4.52102	2.56164	Down
NM_001145350	C20orf173	chromosome 20 open reading frame 173	chr20	-3.05422	3.97112	Down
NM_001009608	C20orf94	chromosome 20 open reading frame 94	chr20	-4.30556	1.7001	Down

NM_001162495	C21orf62	chromosome 21 open reading frame 62	chr21	-4.99723	0.980578	Down
NM_001184958	C3orf20	chromosome 3 open reading frame 20	chr3	-3.06127	4.09374	Down
NM_001184957	C3orf20	chromosome 3 open reading frame 20	chr3	-3.10332	4.17785	Down
NM_032137	C3orf20	chromosome 3 open reading frame 20	chr3	-3.70449	2.58373	Down
NM_130848	C5orf20	TRAF-interacting protein with forkhead-associated domain, family member B; chromosome 5 open reading frame 20	chr5	-3.6345	2.44579	Down
NM_022483	C5orf28	chromosome 5 open reading frame 28	chr5	-3.7931	1.98167	Down
NR_027906	C6orf124	chromosome 6 open reading frame 124	chr6	-3.55105	4.96478	Down
NM_001031743	C6orf165	chromosome 6 open reading frame 165	chr6	-2.55361	2.0335	Down
NM_001033564	C6orf225	chromosome 6 open reading frame 225	chr6	-4.808	0.256836	Down
NM_001127365	C7orf46	chromosome 7 open reading frame 46	chr7	-4.52416	1.47887	Down
NR_026785	C8orf51	chromosome 8 open reading frame 51	chr8	-3.03082	3.83148	Down
NM_001012446	C9orf128	chromosome 9 open reading frame 128	chr9	-2.35135	1.90625	Down
NM_152786	C9orf43	chromosome 9 open reading frame 43	chr9	-5.1097	1.03237	Down
NM_153710	C9orf96	chromosome 9 open reading frame 96	chr9	-4.62211	0.20782	Down
NM_001100619	CABLES1	Cdk5 and Abl enzyme substrate 1	chr18	-5.03034	0.244816	Down
NM_138375	CABLES1	Cdk5 and Abl enzyme substrate 1	chr18	-5.11293	0.356383	Down
NR_023359	CABLES1	Cdk5 and Abl enzyme substrate 1	chr18	-5.18372	0.214791	Down
NM_001143962	CAPN8	calpain 8	chr1	-4.70405	1.80757	Down
NM_020785	CC2D2A	coiled-coil and C2 domain containing 2A	chr4	-5.29781	0.380171	Down
NM_001164720	CC2D2A	coiled-coil and C2 domain containing 2A	chr4	-5.38053	0.492002	Down
NM_152775	CCDC110	coiled-coil domain containing 110	chr4	-3.69331	3.01914	Down
NM_181426	CCDC39	coiled-coil domain containing 39	chr3	-3.66261	2.0656	Down
NM_017950	CCDC40	coiled-coil domain containing 40	chr17	-2.53584	3.51382	Down
NM_001243342	CCDC40	coiled-coil domain containing 40	chr17	-2.73775	3.57864	Down
NM_024725	CCDC82	coiled-coil domain containing 82	chr11	-2.2524	1.57342	Down
NM_173556	CCDC83	coiled-coil domain containing 83	chr11	-3.51778	4.56733	Down
NM_002985	CCL5	chemokine (C-C motif) ligand 5	chr17	3.02119	0.773277	Down
NM_057749	CCNE2	cyclin E2	chr8	-3.50577	1.88645	Down
NM_001838	CCR7	chemokine (C-C motif) receptor 7	chr17	1.104	1.23157	Down
NM_016557	CCRL1	chemokine (C-C motif) receptor-like 1	chr3	-3.88185	2.62096	Down
NM_178445	CCRL1	chemokine (C-C motif) receptor-like 1	chr3	-3.95116	2.75959	Down
NM_001142401	CD164	CD164 molecule, sialomucin	chr6	0.930977	1.1313	Down
NM_006016	CD164	CD164 molecule, sialomucin	chr6	0.930977	1.1313	Down
NM_001142402	CD164	CD164 molecule, sialomucin	chr6	0.930147	1.13092	Down
NM_001764	CD1B	CD1b molecule	chr1	-2.48889	3.11771	Down
NM_138806	CD200R1	CD200 receptor 1	chr3	-3.44969	1.87998	Down
NM_170780	CD200R1	CD200 receptor 1	chr3	-3.44969	1.87998	Down
NM_000073	CD3G	CD3g molecule, gamma (CD3-TCR complex)	chr11	-0.374559	1.433	Down
NM_078481	CD97	CD97 molecule	chr19	2.96119	0.321002	Down
NM_001025160	CD97	CD97 molecule	chr19	2.95156	0.31799	Down
NM_001784	CD97	CD97 molecule	chr19	2.94045	0.314542	Down
NM_031891	CDH20	cadherin 20, type 2	chr18	-2.95518	3.49578	Down
NM_001786	CDK1	cell division cycle 2, G1 to S and G2 to M	chr10	-3.77523	2.15273	Down
NM_033379	CDK1	cell division cycle 2, G1 to S and G2 to M	chr10	-3.90519	2.32336	Down
NM_001025077	CELF2	CUG triplet repeat, RNA binding protein 2	chr10	1.85821	0.966481	Down
NM_005507	CFL1	cofilin 1 (non-muscle)	chr11	3.04513	0.647958	Down
NM_015557	CHD5	chromodomain helicase DNA binding protein 5	chr1	-3.17932	3.063	Down
NM_000745	CHRNA5	cholinergic receptor, nicotinic, alpha 5	chr15	-3.42517	3	Down
NR_023312	CIRBP	cold inducible RNA binding protein	chr19	1.39883	1.0736	Down
NR_023313	CIRBP	cold inducible RNA binding protein	chr19	1.39071	1.07382	Down
NM_153610	CMYA5	cardiomyopathy associated 5	chr5	-3.74442	2.56579	Down
NM_015198	COBL	cordon-bleu homolog (mouse)	chr7	-3.14793	2.04561	Down
NM_000494	COL17A1	collagen, type XVII, alpha 1	chr10	-5.03343	0.012531	Down
NM_198148	CPXM2	carboxypeptidase X (M14 family), member 2	chr10	-2.67462	2.27552	Down
NM_032607	CREB3L3	cAMP responsive element binding protein 3-like 3	chr19	-1.87323	2.11025	Down
NR_028024	CREBZF	CREB/ATF bZIP transcription factor	chr11	-1.17206	1.49843	Down
NM_001202475	CRHR2	corticotropin releasing hormone receptor 2	chr7	-1.7532	1.70464	Down

NM_001202483	CRHR2	corticotropin releasing hormone receptor 2	chr7	-1.7532	1.70464	Down
NM_145203	CSNK1A1L	casein kinase 1, alpha 1-like	chr13	-5.42483	0.403167	Down
NM_001909	CTSD	cathepsin D	chr11	2.74776	0.394354	Down
NM_004079	CTSS	cathepsin S	chr1	2.65461	0.806024	Down
NM_001199739	CTSS	cathepsin S	chr1	2.64609	0.805156	Down
NM_001207063	CXADR	coxsackie virus and adenovirus receptor pseudogene 2; coxsackie virus and adenovirus receptor	chr21	-5.58181	0.366165	Down
NM_001207064	CXADR	coxsackie virus and adenovirus receptor pseudogene 2; coxsackie virus and adenovirus receptor	chr21	-5.58181	0.366165	Down
NM_001207065	CXADR	coxsackie virus and adenovirus receptor pseudogene 2; coxsackie virus and adenovirus receptor	chr21	-5.58181	0.366165	Down
NM_001338	CXADR	coxsackie virus and adenovirus receptor pseudogene 2; coxsackie virus and adenovirus receptor	chr21	-5.58181	0.366165	Down
NM_001168298	CXCR2	interleukin 8 receptor, beta	chr2	3.29098	0.539122	Down
NM_134268	CYGB	cytoglobin	chr17	-5.10971	0.828885	Down
NM_032552	DAB2IP	DAB2 interacting protein	chr9	-4.00738	2.42275	Down
NM_001193416	DDX3X	DEAD (Asp-Glu-Ala-Asp) box polypeptide 3, X-linked	chrX	1.16872	1.02673	Down
NM_001356	DDX3X	DEAD (Asp-Glu-Ala-Asp) box polypeptide 3, X-linked	chrX	1.16872	1.02673	Down
NM_001193417	DDX3X	DEAD (Asp-Glu-Ala-Asp) box polypeptide 3, X-linked	chrX	1.16855	1.02659	Down
NM_001204505	DGKH	diacylglycerol kinase, eta	chr13	-5.19457	0.334564	Down
NM_001204506	DGKH	diacylglycerol kinase, eta	chr13	-5.19457	0.334564	Down
NM_014420	DKK4	dickkopf homolog 4 (Xenopus laevis)	chr8	-3.10122	4.06511	Down
NM_181706	DNAJC24	DnaJ (Hsp40) homolog, subfamily C, member 24	chr11	-2.5832	1.83797	Down
NR_034113	DNAJC27-AS1	DNAJC27 antisense RNA 1	chr2	-3.99736	2.72922	Down
NM_003462	DNALI1	dynein, axonemal, light intermediate chain 1	chr1	-4.80957	1.45526	Down
NR_033787	DNM1P41	DNM1 pseudogene 41	chr15	-5.14979	0.220067	Down
NM_001005463	EBF3	early B-cell factor 3	chr10	-2.45457	1.83542	Down
NM_001197295	ECM2	extracellular matrix protein 2, female organ and adipocyte specific	chr9	-2.89568	3.87717	Down
NM_001393	ECM2	extracellular matrix protein 2, female organ and adipocyte specific	chr9	-2.89568	3.87717	Down
NR_027062	EEF1DP3	eukaryotic translation elongation factor 1 delta pseudogene 3	chr13	-3.40641	2.27626	Down
NM_014971	EFR3B	EFR3 homolog B (S. cerevisiae)	chr2	-4.37669	1.72029	Down
NM_022726	ELOVL4	elongation of very long chain fatty acids (FEN1/Elo2, SUR4/Elo3, yeast)-like 4	chr6	-2.44815	3.10524	Down
NM_001172440	ENDOU	26 serine protease	chr12	-3.10274	3.40903	Down
NM_006025	ENDOU	26 serine protease	chr12	-3.10274	3.40903	Down
NM_001172439	ENDOU	26 serine protease	chr12	-3.61744	2.26476	Down
NR_038351	ENO1-AS1	ENO1 antisense RNA 1	chr1	-2.61166	5.22333	Down
NM_006208	ENPP1	ectonucleotide pyrophosphatase/phosphodiesterase 1	chr6	-4.66557	0.309537	Down
NM_173567	EPHX4	epoxide hydrolase 4	chr1	-4.30035	2.21219	Down
NM_001982	ERBB3	v-erb-b2 erythroblastic leukemia viral oncogene homolog 3 (avian)	chr12	-3.49361	3.12084	Down
NM_001135604	ESM1	endothelial cell-specific molecule 1	chr5	-2.46158	3.1415	Down
NM_007036	ESM1	endothelial cell-specific molecule 1	chr5	-2.46158	3.1415	Down
NM_014209	ETV2	ets variant 2	chr19	-5.01817	0.849158	Down
NM_015065	EXPH5	exophilin 5	chr11	-4.61673	1.37516	Down
NM_001077710	FAM110C	family with sequence similarity 110, member C	chr2	-3.28819	3.20753	Down
NM_001079529	FAM153B	family with sequence similarity 153, member B	chr5	-3.20078	1.66493	Down
NM_015091	FAM179B	family with sequence similarity 179, member B	chr14	-2.85753	1.97649	Down
NM_001129891	FAM196B	Uncharacterized protein LOC100131897	chr5	-5.17899	0.617101	Down
NM_001195228	FAM64A	family with sequence similarity 64, member A	chr17	-3.1369	2.30116	Down
NM_019013	FAM64A	family with sequence similarity 64, member A	chr17	-3.1369	2.30116	Down
NR_026789	FAM66A	family with sequence similarity 66, member A	chr8	-3.16405	2.03868	Down
NM_001104545	FAM70A	family with sequence similarity 70, member A	chrX	-3.99895	1.85567	Down
NR_024252	FAM86HP	family with sequence similarity 86, member A pseudogene	chr3	-4.7223	1.79198	Down
NM_001024215	FBLIM1	filamin binding LIM protein 1	chr1	-5.08298	1.64139	Down

NM_001142958	FBXO15	F-box protein 15	chr18	-1.7147	1.91542	Down
NM_152676	FBXO15	F-box protein 15	chr18	-1.7147	1.91542	Down
NM_153230	FBXO39	F-box protein 39	chr17	-3.51565	2.16652	Down
NM_001136219	FCGR2A	Fc fragment of IgG, low affinity IIa, receptor (CD32)	chr1	2.8854	0.662798	Down
NM_021642	FCGR2A	Fc fragment of IgG, low affinity IIa, receptor (CD32)	chr1	2.88539	0.662776	Down
NM_004108	FCN2	ficolin (collagen/fibrinogen domain containing lectin) 2 (hucolin)	chr9	-4.73911	1.72644	Down
NM_015837	FCN2	ficolin (collagen/fibrinogen domain containing lectin) 2 (hucolin)	chr9	-4.73911	1.72644	Down
NM_005246	FER	fer (fps/fes related) tyrosine kinase	chr5	-4.2922	2.22889	Down
NM_001134999	FERMT2	fermitin family homolog 2 (Drosophila)	chr14	-2.07499	4.14999	Down
NM_006832	FERMT2	fermitin family homolog 2 (Drosophila)	chr14	-2.07499	4.14999	Down
NR_026655	FLJ12825	hypothetical LOC440101	chr12	-3.15762	2.53677	Down
NR_039985	FLJ22447	hypothetical gene supported by AK026100	chr14	-3.12874	4.18917	Down
NR_029434	FLJ31306	hypothetical LOC379025	chr14	-0.162202	1.36797	Down
NR_029435	FLJ31306	hypothetical LOC379025	chr14	-0.18035	1.36982	Down
NR_015392	FLJ40852	hypothetical LOC285962	chr7	-2.60851	2.27778	Down
NR_028139	FLJ42289	hypothetical LOC388182	chr15	-4.3236	1.65802	Down
NM_207414	FLJ43860	FLJ43860 protein	chr8	-3.75382	3.38005	Down
NR_028337	FLJ45079	FLJ45079 protein	chr17	-4.10771	2.7607	Down
NM_001110556	FLNA	filamin A, alpha (actin binding protein 280)	chrX	3.74377	0.35855	Down
NM_001456	FLNA	filamin A, alpha (actin binding protein 280)	chrX	3.74376	0.358548	Down
NR_034096	FONG	formiminotransferase cyclodeaminase N-terminal like	chr2	-2.69475	2.31579	Down
NM_005252	FOS	v-fos FBJ murine osteosarcoma viral oncogene homolog	chr14	1.7886	0.0649187	Down
NM_207305	FOXD4	forkhead box D4	chr9	-5.53458	0.0682894	Down
NR_024006	FP588	hypothetical LOC92973	chr9	-4.98531	0.942699	Down
NM_173651	FSIP2	fibrous sheath interacting protein 2	chr2	-2.45167	2.67318	Down
NM_015082	FSTL4	follistatin-like 4	chr5	-2.48234	1.68519	Down
NM_031866	FZD8	frizzled homolog 8 (Drosophila)	chr10	-3.2161	2.14277	Down
NM_005256	GAS2	growth arrest-specific 2	chr11	-2.94411	3.55238	Down
NM_177553	GAS2	growth arrest-specific 2	chr11	-2.94411	3.55238	Down
NM_001143830	GAS2	growth arrest-specific 2	chr11	-3.57123	2.29815	Down
NM_174942	GAS2L3	growth arrest-specific 2 like 3	chr12	-3.15951	4.12803	Down
NR_003267	GGT3P	gamma-glutamyltransferase 3 pseudogene	chr22	-5.54771	0.872603	Down
NM_018384	GIMAP5	GTPase, IMAP family member 5	chr7	0.826704	1.18998	Down
NM_015710	GLTSCR2	glioma tumor suppressor candidate region gene 2; glioma tumor suppressor candidate region gene 2 pseudogene	chr19	2.08926	0.956044	Down
NM_002069	GNAI1	guanine nucleotide binding protein (G protein), alpha inhibiting activity polypeptide 1	chr7	-3.80218	2.80029	Down
NM_002075	GNB3	guanine nucleotide binding protein (G protein), beta polypeptide 3	chr12	-4.73989	1.96127	Down
NM_001501	GNRH2	gonadotropin-releasing hormone 2	chr20	-2.55948	2.04524	Down
NM_178331	GNRH2	gonadotropin-releasing hormone 2	chr20	-2.55948	2.04524	Down
NM_178332	GNRH2	gonadotropin-releasing hormone 2	chr20	-2.55948	2.04524	Down
NM_001001413	GOLGA6L1	hypothetical LOC645202; Putative golgin subfamily A member 6-like protein 6; hypothetical LOC100132202; golgi autoantigen, golgin subfamily a-like	chr15	-5.3915	1.0975	Down
NM_001145004	GOLGA6L6	hypothetical LOC645202; Putative golgin subfamily A member 6-like protein 6; hypothetical LOC100132202; golgi autoantigen, golgin subfamily a-like	chr15	-5.32261	1.23527	Down
NM_181077	GOLGA8A	golgi autoantigen, golgin subfamily a, 8B; golgi autoantigen, golgin subfamily a, 8A	chr15	0.00350862	1.32592	Down
NM_001023567	GOLGA8B	golgi autoantigen, golgin subfamily a, 8B; golgi autoantigen, golgin subfamily a, 8A	chr15	-0.0712312	1.42208	Down
NM_000407	GP1BB	glycoprotein Ib (platelet), beta polypeptide	chr22	1.33414	0.31701	Down
NM_199243	GPR150	G protein-coupled receptor 150	chr5	-4.38755	1.9966	Down
NM_018969	GPR173	G protein-coupled receptor 173	chrX	-4.37991	2.28779	Down
NM_031936	GPR61	G protein-coupled receptor 61	chr1	-2.04078	1.61333	Down
NM_001191015	GSTO2	glutathione S-transferase omega 2	chr10	-4.42727	1.96967	Down
NR_001317	HCG4B	HLA complex group 4 pseudogene 6	chr6	-1.74209	1.72686	Down

NM_173497	HECTD2	HECT domain containing 2	chr10	-2.41831	2.69929	Down
NM_182765	HECTD2	HECT domain containing 2	chr10	-3.3386	2.81036	Down
NM_001098672	HEPHL1	hephaestin-like 1	chr11	-3.96639	2.62785	Down
NM_005320	HIST1H1D	histone cluster 1, H1d	chr6	-3.00173	2.2987	Down
NM_003535	HIST1H3J	histone cluster 1, H3j; histone cluster 1, H3i; histone cluster 1, H3h; histone cluster 1, H3g; histone cluster 1, H3f; histone cluster 1, H3e; histone cluster 1, H3d; histone cluster 1, H3c; histone cluster 1, H3b; histone cluster 1, H3a; histone cluster 1, H2ad; histone cluster 2, H3a; histone cluster 2, H3c; histone cluster [illegible]	chr6	-3.66319	3.56017	Down
NM_001005464	HIST2H3A	histone cluster 1, H3j; histone cluster 1, H3i; histone cluster 1, H3h; histone cluster 1, H3g; histone cluster 1, H3f; histone cluster 1, H3e; histone cluster 1, H3d; histone cluster 1, H3c; histone cluster 1, H3b; histone cluster 1, H3a; histone cluster 1, H2ad; histone cluster 2, H3a; histone cluster 2, H3c; histone cluster [illegible]	chr1	-4.62379	0.216835	Down
NM_021059	HIST2H3C	histone cluster 1, H3j; histone cluster 1, H3i; histone cluster 1, H3h; histone cluster 1, H3g; histone cluster 1, H3f; histone cluster 1, H3e; histone cluster 1, H3d; histone cluster 1, H3c; histone cluster 1, H3b; histone cluster 1, H3a; histone cluster 1, H2ad; histone cluster 2, H3a; histone cluster 2, H3c; histone cluster [illegible]	chr1	-4.62379	0.216835	Down
NR_001435	HLA-DPB2	major histocompatibility complex, class II, DP beta 2 (pseudogene)	chr6	-3.6645	2.47396	Down
NM_002126	HLF	hepatic leukemia factor	chr17	-4.65708	1.49907	Down
NM_012292	HMHA1	histocompatibility (minor) HA-1	chr19	2.64741	0.612418	Down
NM_031243	HNRNPA2B1	heterogeneous nuclear ribonucleoprotein A2/B1	chr7	1.23687	1.06176	Down
NM_002137	HNRNPA2B1	heterogeneous nuclear ribonucleoprotein A2/B1	chr7	1.2362	1.06241	Down
NM_005520	HNRNPH1	heterogeneous nuclear ribonucleoprotein H1 (H)	chr5	0.950362	1.16838	Down
NM_019102	HOXA5	homeobox A5	chr7	-2.86028	5.72055	Down
NM_032756	HPDL	4-hydroxyphenylpyruvate dioxygenase-like	chr1	-4.7552	0.320091	Down
NM_014485	HPGDS	prostaglandin D2 synthase, hematopoietic; prostaglandin D2 synthase 21kDa (brain)	chr4	-3.49452	3.13905	Down
NM_144617	HSPB6	heat shock protein, alpha-crystallin-related, B6	chr19	-5.30244	0.720962	Down
NM_000867	HTR2B	5-hydroxytryptamine (serotonin) receptor 2B	chr2	-2.48204	3.01065	Down
NM_178231	ICA1L	islet cell autoantigen 1,69kDa-like	chr2	-3.69476	1.80734	Down
NM_002162	ICAM3	intercellular adhesion molecule 3	chr19	2.79735	0.430351	Down
NM_021034	IFITM3	interferon induced transmembrane protein 3 (1-8U)	chr11	3.03541	0.352729	Down
NM_031473	IFT81	intraflagellar transport 81 homolog (Chlamydomonas)	chr12	-5.08331	0.0916063	Down
NR_002937	IGBP1P1	chromosome 14 open reading frame 19; immunoglobulin (CD79A) binding protein 1	chr14	-3.29633	4.73259	Down
NM_020962	IGDCC4	immunoglobulin superfamily, DCC subclass, member 4	chr15	-2.46889	4.93778	Down
NM_001015887	IGSF11	immunoglobulin superfamily, member 11	chr3	-2.64947	3.34552	Down
NM_000572	IL10	interleukin 10	chr1	-4.49037	1.979	Down
NM_014443	IL17B	interleukin 17B	chr5	-2.55122	3.23817	Down
NM_000586	IL2	interleukin 2	chr4	-2.74625	2.27324	Down
NM_018724	IL20	interleukin 20	chr1	-2.02256	4.04513	Down
NM_144701	IL23R	interleukin 23 receptor	chr1	-2.11791	2.73123	Down
NM_001012633	IL32	interleukin 32	chr16	2.02039	1.14679	Down
NM_000589	IL4	interleukin 4	chr5	-3.3523	3.48316	Down
NM_172348	IL4	interleukin 4	chr5	-3.3523	3.48316	Down
NM_000600	IL6	interleukin 6 (interferon, beta 2)	chr7	-1.96698	2.25779	Down
NM_002184	IL6ST	interleukin 6 signal transducer (gp130, oncostatin M receptor)	chr5	-0.291248	1.54706	Down
NM_175767	IL6ST	interleukin 6 signal transducer (gp130, oncostatin M receptor)	chr5	-0.292039	1.54864	Down
NM_001190981	IL6ST	interleukin 6 signal transducer (gp130, oncostatin M receptor)	chr5	-0.296793	1.54731	Down
NM_002185	IL7R	interleukin 7 receptor	chr5	0.438328	1.47532	Down
NM_001199800	ILDR1	immunoglobulin-like domain containing receptor 1	chr3	-2.88636	2.56632	Down
NM_199351	ILDR2	immunoglobulin-like domain containing receptor 2	chr1	-4.8173	1.89495	Down

NM_032727	INA	internexin neuronal intermediate filament protein, alpha	chr10	-2.10816	2.30213	Down
NR_037598	INMT-FAM18	INMT-FAM188B readthrough (NMD candidate)	chr7	-5.11285	0.634966	Down
NM_024726	IQCA1	IQ motif containing with AAA domain 1	chr2	-2.77183	3.67939	Down
NM_015232	IQSEC3	IQ motif and Sec7 domain 3; similar to IQ motif and Sec7 domain-containing protein 3; similar to IQ motif and SEC7 domain-containing protein 3; similar to IQ motif and Sec7 domain 3	chr12	-3.53873	4.11287	Down
NM_000885	ITGA4	integrin, alpha 4 (antigen CD49D, alpha 4 subunit of VLA-4 receptor)	chr2	0.642639	1.27928	Down
NM_002207	ITGA9	integrin, alpha 9	chr3	-5.20127	0.57203	Down
NM_000887	ITGAX	integrin, alpha X (complement component 3 receptor 4 subunit)	chr16	2.75068	0.530054	Down
NM_002220	ITPKA	inositol 1,4,5-trisphosphate 3-kinase A	chr15	-3.29384	2.58807	Down
NM_020122	KCMF1	potassium channel modulatory factor 1	chr2	0.250834	0.133695	Down
NM_021161	KCNK10	potassium channel, subfamily K, member 10	chr14	-3.6641	2.39443	Down
NM_138317	KCNK10	potassium channel, subfamily K, member 10	chr14	-3.6641	2.39443	Down
NM_138318	KCNK10	potassium channel, subfamily K, member 10	chr14	-3.6641	2.39443	Down
NM_001080392	KIAA1147	KIAA1147	chr7	-0.786557	1.40753	Down
NM_001122819	KIF17	kinesin family member 17	chr1	-1.86423	1.97662	Down
NM_020816	KIF17	kinesin family member 17	chr1	-1.86423	1.97662	Down
NM_001161707	KIRREL3	kin of IRRE like 3 (Drosophila)	chr11	-2.34835	2.7825	Down
NM_004795	KL	klotho	chr13	-2.8033	3.65318	Down
NM_020805	KLHL14	kelch-like 14 (Drosophila)	chr18	-2.80002	1.77944	Down
NM_002269	KPNA5	karyopherin alpha 5 (importin alpha 6)	chr6	-3.81312	1.86147	Down
NM_002281	KRT81	keratin 81	chr12	-2.53571	3.28976	Down
NM_001005922	KRTAP5-1	keratin associated protein 5-1	chr11	-3.43254	2.24263	Down
NM_001004325	KRTAP5-2	keratin associated protein 5-2	chr11	-3.44052	3.15681	Down
NM_018490	LGR4	leucine-rich repeat-containing G protein-coupled receptor 4	chr11	-2.73139	1.98158	Down
NM_030576	LIMD2	LIM domain containing 2	chr17	2.65417	0.62414	Down
NR_027266	LINC00310	chromosome 21 open reading frame 82	chr21	-2.10991	4.21982	Down
NM_001128215	LIPM	lipase, family member M	chr10	-4.54119	2.08011	Down
NM_001085451	LNP1	leukemia NUP98 fusion partner 1	chr3	-3.1308	2.61556	Down
NR_027274	LOC10012878	hypothetical protein LOC100128788	chr16	-5.41586	0.63564	Down
NM_001195279	LOC10012948	similar to hCG2042446	chr3	-4.64384	0.301539	Down
NR_038910	LOC10012984	hypothetical LOC100129845	chr7	-2.95205	2.34222	Down
NR_033939	LOC10012985	hypothetical protein LOC100129858	chr4	-4.34455	1.91918	Down
NM_001242750	LOC10012992	similar to hCG2036949	chr1	-5.08361	2.07804	Down
NR_038446	LOC10013023	similar to hCG1814455	chr8	0.832748	1.4686	Down
NR_034016	LOC10013095	similar to hCG2021566	chr9	-4.56686	1.91505	Down
NR_034017	LOC10013095	similar to hCG2021566	chr9	-4.56686	1.91505	Down
NR_026658	LOC10024073	hypothetical LOC100240735	chr12	-4.71563	1.54331	Down
NR_038390	LOC10028807	similar to hCG2036779	chr11	-1.85445	1.83305	Down
NR_034101	LOC10028834	hypothetical protein LOC100288346	chr11	-5.21112	1.09101	Down
NR_036530	LOC10028923	hypothetical protein LOC100289230	chr5	-2.64932	1.88137	Down
NR_036507	LOC10030697	NADH dehydrogenase (ubiquinone) complex I, assembly factor 4 pseudogene 1	chr15	-2.70152	4.06071	Down
NR_038340	LOC10050581	uncharacterized LOC100505817	chr18	-3.09358	3.77257	Down
NR_038949	LOC10050619	LARGE antisense RNA 1	chr22	-2.9031	3.39161	Down
NR_036592	LOC10050671	endogenous Bornavirus-like nucleoprotein 2 pseudogene	chr9	-0.513119	1.46326	Down
NR_038323	LOC10050715	uncharacterized LOC100507156	chr8	-3.45819	2.16596	Down
NR_038293	LOC10050717	long intergenic non-protein coding RNA 1012	chr6	-2.12032	4.24065	Down
NR_038294	LOC10050717	long intergenic non-protein coding RNA 1012	chr6	-2.12032	4.24065	Down
NR_038987	LOC10050748	uncharacterized LOC100507489	chr6	-2.08514	4.17029	Down
NR_033252	LOC145663	hypothetical protein LOC145663	chr15	-2.53071	1.98771	Down
NR_038222	LOC219731	hypothetical protein LOC219731	chr10	-3.21375	2.06002	Down
NR_039982	LOC283547	hypothetical protein LOC283547	chr14	-2.89724	3.72616	Down
NR_027085	LOC284551	hypothetical LOC284551	chr1	-3.21293	3.6294	Down
NR_026968	LOC285456	hypothetical LOC285456	chr4	-3.50564	4.5028	Down
NR_037863	LOC285484	hypothetical protein LOC285484	chr4	-3.68418	2.80061	Down
NR_027108	LOC285593	hypothetical LOC285593	chr5	-3.87673	2.76603	Down

NR_026975	LOC286467	hypothetical LOC286467	chrX	-2.94462	2.27333	Down
NR_021489	LOC338651	hypothetical protein LOC338651	chr11	-2.86189	2.23466	Down
NR_033890	LOC338817	hypothetical protein LOC338817	chr12	-2.77483	2.43652	Down
NR_026989	LOC339524	hypothetical LOC339524	chr1	-2.78594	3.5126	Down
NR_038990	LOC339926	hypothetical LOC339926	chr3	-2.78746	3.5066	Down
NR_034099	LOC388948	hypothetical protein LOC388948	chr2	-2.47844	4.95688	Down
NR_033995	LOC389247	hypothetical gene supported by BC038563	chr4	-3.78033	2.73542	Down
NR_024425	LOC389791	hypothetical LOC389791	chr9	-3.04142	1.93844	Down
NM_001193282	LOC402160	similar to hCG18094	chr4	-2.98118	3.32809	Down
NR_036683	LOC440700	carbonic anhydrase XIV (CA14) pseudogene	chr1	-2.66868	5.33737	Down
NR_026792	LOC441455	makorin ring finger protein 1 pseudogene	chr9	-2.7961	2.79575	Down
NR_024380	LOC441666	zinc finger protein 91 pseudogene	chr10	-2.61788	3.17648	Down
NR_024496	LOC442421	hypothetical LOC442421	chr9	-3.02661	3.82306	Down
NR_040096	LOC643339	similar to nascent polypeptide-associated complex alpha subunit	chr12	-3.22268	2.94763	Down
NR_038848	LOC643401	hypothetical protein LOC643401	chr5	-2.1057	2.23626	Down
NR_033921	LOC643542	hypothetical protein LOC643542	chr18	-2.4905	3.01577	Down
NR_027620	LOC643719	hypothetical LOC643719	chr19	-4.36287	2.16017	Down
NR_030732	LOC645638	similar to WDNM1-like protein	chr17	-4.10265	2.4244	Down
NR_027374	LOC723809	hypothetical LOC723809	chr7	-3.39417	4.65101	Down
NR_024403	LOC730101	hypothetical LOC730101	chr6	-2.89083	1.85363	Down
NR_024405	LOC730101	hypothetical LOC730101	chr6	-2.90907	1.81716	Down
NR_038421	LOC731223	hypothetical LOC731223	chr14	-3.07163	2.27563	Down
NR_026794	LOC731789	hypothetical LOC731789	chr10	-2.73339	2.67033	Down
NM_144648	LRGUK	leucine-rich repeats and guanylate kinase domain containing	chr7	-4.84279	1.84367	Down
NM_001161575	LRRC36	leucine rich repeat containing 36	chr16	-3.46452	3.19395	Down
NM_018296	LRRC36	leucine rich repeat containing 36	chr16	-3.46452	3.19395	Down
NM_006107	LUC7L3	cisplatin resistance-associated overexpressed protein	chr17	0.212703	1.40496	Down
NM_020426	LYZL6	lysozyme-like 6	chr17	-1.98861	2.2846	Down
NM_005921	MAP3K1	mitogen-activated protein kinase kinase kinase 1	chr5	0.160536	1.28428	Down
NM_021960	MCL1	myeloid cell leukemia sequence 1 (BCL2-related)	chr1	2.76353	0.817991	Down
NM_182763	MCL1	myeloid cell leukemia sequence 1 (BCL2-related)	chr1	2.741	0.811328	Down
NM_001197320	MCL1	myeloid cell leukemia sequence 1 (BCL2-related)	chr1	2.68983	0.82299	Down
NM_001166345	MDFIC	MyoD family inhibitor domain containing	chr7	-1.22543	1.64753	Down
NM_199072	MDFIC	MyoD family inhibitor domain containing	chr7	-1.22543	1.64753	Down
NM_001199172	MGAT5B	mannosyl (alpha-1,6-)-glycoprotein beta-1,6-N-acetyl-glucosaminyltransferase, isozyme B	chr17	-3.29748	4.36479	Down
NM_144677	MGAT5B	mannosyl (alpha-1,6-)-glycoprotein beta-1,6-N-acetyl-glucosaminyltransferase, isozyme B	chr17	-3.29748	4.36479	Down
NM_198955	MGAT5B	mannosyl (alpha-1,6-)-glycoprotein beta-1,6-N-acetyl-glucosaminyltransferase, isozyme B	chr17	-3.29748	4.36479	Down
NR_031593	MIR1182	microRNA 1182	chr1	-5.51863	0.492347	Down
NR_031602	MIR1237	microRNA 1237	chr11	-2.37487	2.4139	Down
NR_031603	MIR1238	microRNA 1238	chr19	-5.50018	1.08273	Down
NR_031619	MIR1287	microRNA 1287	chr10	-4.05506	4.13515	Down
NR_027180	MIR143HG	hypothetical LOC728264	chr5	-1.92575	2.29852	Down
NR_029518	MIR29B2	microRNA 29b-2	chr1	-4.53874	1.39686	Down
NR_029858	MIR302C	microRNA 302c	chr4	-3.17487	3.84128	Down
NR_037412	MIR3618	microRNA 3618	chr22	-5.12774	1.15836	Down
NR_037426	MIR3653	microRNA 3653	chr22	-3.66167	2.47902	Down
NR_037448	MIR3677	microRNA 3677	chr16	-3.33409	4.33233	Down
NR_037500	MIR3936	microRNA 3936	chr5	-2.80493	3.74979	Down
NR_030398	MIR421	microRNA 421	chrX	-5.32402	1.23135	Down
NR_029948	MIR425	microRNA 425	chr3	-3.53856	2.14334	Down
NR_036198	MIR4313	microRNA 4313	chr15	-2.33489	2.6105	Down
NR_039644	MIR4442	microRNA 4442	chr3	-5.13686	0.701026	Down
NR_039658	MIR4453	microRNA 4453	chr4	-3.12679	4.0626	Down
NR_039778	MIR4635	microRNA 4635	chr5	-3.11309	4.27276	Down
NR_039788	MIR4645	microRNA 4645	chr6	-4.46821	2.17944	Down

NR_039790	MIR4647	microRNA 4647	chr6	-4.41891	1.72379	Down
NR_039862	MIR4712	microRNA 4712	chr15	-4.72583	0.227936	Down
NR_039924	MIR4767	microRNA 4767	chrX	-2.92875	2.78379	Down
NR_039946	MIR4785	microRNA 4785	chr2	-4.77715	1.97427	Down
NR_039949	MIR4786	microRNA 4786	chr2	-2.38669	2.94507	Down
NR_039956	MIR4793	microRNA 4793	chr3	-2.10546	1.79634	Down
NR_039969	MIR5047	microRNA 5047	chr17	-1.83272	1.69521	Down
NR_030282	MIR555	microRNA 555	chr1	-4.81421	1.18442	Down
NR_030290	MIR564	microRNA 564	chr3	-5.2351	0.505362	Down
NR_030367	MIR637	microRNA 637	chr19	-5.20079	1.12699	Down
NR_030372	MIR642A	microRNA 642a	chr19	-2.72635	2.06229	Down
NR_037512	MIR642B	microRNA 642b	chr19	-2.73972	2.08904	Down
NR_030375	MIR645	microRNA 645	chr20	-5.20666	0.0807096	Down
NR_030633	MIR937	microRNA 937	chr8	-5.78315	0.239882	Down
NM_002421	MMP1	matrix metallopeptidase 1 (interstitial collagenase)	chr11	-3.08417	2.73809	Down
NM_001145938	MMP1	matrix metallopeptidase 1 (interstitial collagenase)	chr11	-3.09371	2.71901	Down
NM_022782	MPHOSPH9	M-phase phosphoprotein 9	chr12	-2.0683	1.64916	Down
NM_002444	MSN	moesin	chrX	3.35601	0.582886	Down
NM_001080416	MYBL1	v-myb myeloblastosis viral oncogene homolog (avian)-like 1	chr8	-1.3257	1.62294	Down
NM_001144755	MYBL1	v-myb myeloblastosis viral oncogene homolog (avian)-like 1	chr8	-1.34854	1.62029	Down
NM_016132	MYEF2	myelin expression factor 2	chr15	-3.24403	2.02381	Down
NM_002473	MYH9	myosin, heavy chain 9, non-muscle	chr22	3.47971	0.455864	Down
NM_001101421	MYO1H	myosin IH	chr12	-3.12346	4.27178	Down
NM_015460	MYRIP	myosin VIIA and Rab interacting protein	chr3	-2.1517	1.79493	Down
NM_000265	NCF1	neutrophil cytosolic factor 1; neutrophil cytosolic factor 1C pseudogene	chr7	3.16955	0.386577	Down
NR_003186	NCF1B	neutrophil cytosolic factor 1B pseudogene	chr7	2.6366	0.409323	Down
NR_003187	NCF1C	neutrophil cytosolic factor 1; neutrophil cytosolic factor 1C pseudogene	chr7	2.61806	0.373466	Down
NM_001173484	NEBL	nebulette	chr10	-2.6301	5.2602	Down
NM_213569	NEBL	nebulette	chr10	-2.6301	5.2602	Down
NM_006393	NEBL	nebulette	chr10	-2.67059	5.34118	Down
NM_001204182	NEK2	NIMA (never in mitosis gene a)-related kinase 2	chr1	-2.66415	2.8091	Down
NM_001204183	NEK2	NIMA (never in mitosis gene a)-related kinase 2	chr1	-2.66415	2.8091	Down
NM_002497	NEK2	NIMA (never in mitosis gene a)-related kinase 2	chr1	-3.65357	2.59696	Down
NM_004289	NFE2L3	nuclear factor (erythroid-derived 2)-like 3	chr7	-2.36052	1.70728	Down
NM_001190737	NFIB	nuclear factor I/B	chr9	-2.41865	1.92264	Down
NM_001190738	NFIB	nuclear factor I/B	chr9	-2.43043	1.94621	Down
NM_005596	NFIB	nuclear factor I/B	chr9	-2.43043	1.94621	Down
NM_005601	NKG7	natural killer cell group 7 sequence	chr19	2.52587	0.822213	Down
NM_014893	NLGN4Y	neuroligin 4, Y-linked	chrY	-3.45987	2.64088	Down
NR_028319	NLGN4Y	neuroligin 4, Y-linked	chrY	-3.45987	2.64088	Down
NM_001164757	NOS1AP	nitric oxide synthase 1 (neuronal) adaptor protein	chr1	-3.31028	2.73436	Down
NM_014697	NOS1AP	nitric oxide synthase 1 (neuronal) adaptor protein	chr1	-3.31028	2.73436	Down
NM_001204376	NPR3	natriuretic peptide receptor C/guanylate cyclase C (atrionatriuretic peptide receptor C)	chr5	-2.57134	2.25933	Down
NM_033013	NR1I2	nuclear receptor subfamily 1, group I, member 2	chr3	-2.62403	2.77982	Down
NM_016588	NRN1	neuritin 1	chr6	-4.31022	1.70569	Down
NM_021229	NTN4	netrin 4	chr12	-3.9775	2.35984	Down
NM_022731	NUCKS1	nuclear casein kinase and cyclin-dependent kinase substrate 1	chr1	0.0559201	1.27598	Down
NR_002779	NUDT9P1	nudix (nucleoside diphosphate linked moiety X)-type motif 9 pseudogene 1	chr10	-3.30054	2.17994	Down
NM_001173431	OBSL1	obscurin-like 1	chr2	-3.82256	2.32053	Down
NM_015311	OBSL1	obscurin-like 1	chr2	-3.82256	2.32053	Down
NM_022375	OCLM	oculomedin	chr1	-3.76007	2.67583	Down
NM_006188	OCM2	oncomodulin 2	chr7	-3.56903	2.44474	Down
NM_053280	ODF3	outer dense fiber of sperm tails 3	chr11	-3.17345	2.2498	Down
NM_014057	OGN	osteoglycin	chr9	-2.26379	1.73113	Down

NM_033014	OGN	osteoglycin	chr9	-2.26379	1.73113	Down
NM_181672	OGT	O-linked N-acetylglucosamine (GlcNAc) transferase (UDP-N-acetylglucosamine:polypeptide-N-acetylglucosaminyl transferase)	chrX	1.31002	1.2862	Down
NM_181673	OGT	O-linked N-acetylglucosamine (GlcNAc) transferase (UDP-N-acetylglucosamine:polypeptide-N-acetylglucosaminyl transferase)	chrX	1.30997	1.28609	Down
NM_001004297	OR13A1	olfactory receptor, family 13, subfamily A, member 1	chr10	-3.32792	2.67384	Down
NR_002163	OR7E37P	olfactory receptor, family 7, subfamily E, member 37 pseudogene	chr13	-5.12447	1.44251	Down
NM_206837	OSCP1	chromosome 1 open reading frame 102	chr1	-4.46876	2.75392	Down
NM_145047	OSCP1	chromosome 1 open reading frame 102	chr1	-4.90805	1.71345	Down
NM_000915	OXT	oxytocin, prepropeptide	chr20	-4.04944	2.37521	Down
NM_207421	PADI6	peptidyl arginine deiminase, type VI	chr1	-5.10682	0.117823	Down
NM_004562	PARK2	Parkinson disease (autosomal recessive, juvenile) 2, parkin	chr6	-5.25188	1.14034	Down
NM_013987	PARK2	Parkinson disease (autosomal recessive, juvenile) 2, parkin	chr6	-5.25188	1.14034	Down
NR_022011	PAR-SN	paternally expressed transcript PAR-SN	chr15	-3.71797	1.82843	Down
NM_018222	PARVA	parvin, alpha	chr11	-3.74249	1.97902	Down
NM_020524	PBXIP1	pre-B-cell leukemia homeobox interacting protein 1	chr1	1.94658	0.324556	Down
NM_018945	PDE7B	phosphodiesterase 7B	chr6	-4.57111	1.55575	Down
NM_002619	PF4	platelet factor 4	chr4	1.25465	0.156566	Down
NM_152595	PGBD4	piggyBac transposable element derived 4	chr15	-4.10254	2.33051	Down
NM_001144758	PHLDB1	pleckstrin homology-like domain, family B, member 1	chr11	-3.78435	2.24463	Down
NM_001144759	PHLDB1	pleckstrin homology-like domain, family B, member 1	chr11	-3.78435	2.24463	Down
NM_015157	PHLDB1	pleckstrin homology-like domain, family B, member 1	chr11	-3.78435	2.24463	Down
NM_001199287	PHOSPHO2	phosphatase, orphan 2	chr2	-4.63194	1.34546	Down
NM_001199285	PHOSPHO2	phosphatase, orphan 2	chr2	-4.87395	1.50493	Down
NM_181794	PKIB	protein kinase (cAMP-dependent, catalytic) inhibitor beta	chr6	-3.55635	2.36334	Down
NM_003561	PLA2G10	phospholipase A2, group X	chr16	-2.90113	2.03605	Down
NM_000930	PLAT	plasminogen activator, tissue	chr8	-4.86487	1.5759	Down
NM_033011	PLAT	plasminogen activator, tissue	chr8	-4.86487	1.5759	Down
NM_001165979	PLCE1	phospholipase C, epsilon 1	chr10	-3.64366	2.95481	Down
NM_016341	PLCE1	phospholipase C, epsilon 1	chr10	-3.64366	2.95481	Down
NM_020715	PLEKHH1	pleckstrin homology domain containing, family H (with MyTH4 domain) member 1	chr14	-3.85015	1.96822	Down
NM_172069	PLEKHH2	pleckstrin homology domain containing, family H (with MyTH4 domain) member 2	chr2	-2.35625	2.75907	Down
NM_001200053	PMEL	premelanosome protein	chr12	-4.37225	1.7289	Down
NM_001144032	PPIAL4E	peptidylprolyl isomerase A (cyclophilin A)-like 4E; peptidylprolyl isomerase A (cyclophilin A)-like 4G; peptidylprolyl isomerase A (cyclophilin A)-like 4F; peptidylprolyl isomerase A (cyclophilin A)-like 4A; peptidylprolyl isomerase A (cyclophilin A)-like 4C; peptidylprolyl isomerase A (cyclophilin A)-like 4B	chr1	-2.55848	3.04864	Down
NM_001123068	PPIAL4G	peptidylprolyl isomerase A (cyclophilin A)-like 4E; peptidylprolyl isomerase A (cyclophilin A)-like 4G; peptidylprolyl isomerase A (cyclophilin A)-like 4F; peptidylprolyl isomerase A (cyclophilin A)-like 4A; peptidylprolyl isomerase A (cyclophilin A)-like 4C; peptidylprolyl isomerase A (cyclophilin A)-like 4B	chr1	-4.04952	1.83481	Down
NM_017726	PPP1R14D	protein phosphatase 1, regulatory (inhibitor) subunit 14D	chr15	-2.51879	1.96387	Down
NM_001007533	PPP1R27	dysferlin interacting protein 1	chr17	-3.32515	4.51297	Down
NM_012094	PRDX5	peroxiredoxin 5	chr11	0.932033	0.140472	Down
NM_181651	PRDX5	peroxiredoxin 5	chr11	0.914244	0.136046	Down
NM_212471	PRKAR1A	protein kinase, cAMP-dependent, regulatory, type I, alpha (tissue specific extinguisher 1)	chr17	0.72567	1.12365	Down
NM_022119	PRSS22	protease, serine, 22	chr16	-2.78797	3.34578	Down
NM_001042465	PSAP	prosaposin	chr10	3.93909	0.39576	Down

NM_001042466	PSAP	prosaposin	chr10	3.93907	0.395644	Down
NM_002778	PSAP	prosaposin	chr10	3.93902	0.395681	Down
NM_001128217	PSIP1	PC4 and SFRS1 interacting protein 1	chr9	0.00364619	1.30426	Down
NM_033222	PSIP1	PC4 and SFRS1 interacting protein 1	chr9	0.00364619	1.30426	Down
NM_176783	PSME1	proteasome (prosome, macropain) activator subunit 1 (PA28 alpha)	chr14	1.82666	0.423353	Down
NM_006263	PSME1	proteasome (prosome, macropain) activator subunit 1 (PA28 alpha)	chr14	1.82546	0.422334	Down
NM_001099666	PTAR1	protein prenyltransferase alpha subunit repeat containing 1	chr9	-1.02704	1.57217	Down
NM_001099285	PTMA	hypothetical LOC728026; prothymosin, alpha; hypothetical gene supported by BC013859; prothymosin, alpha pseudogene 4 (gene sequence 112)	chr2	2.65382	0.931341	Down
NM_002823	PTMA	hypothetical LOC728026; prothymosin, alpha; hypothetical gene supported by BC013859; prothymosin, alpha pseudogene 4 (gene sequence 112)	chr2	2.65379	0.931331	Down
NM_000448	RAG1	recombination activating gene 1	chr11	-2.25825	2.72545	Down
NM_001172732	RAI2	retinoic acid induced 2	chrX	-5.08465	0.758442	Down
NM_021785	RAI2	retinoic acid induced 2	chrX	-5.08465	0.758442	Down
NR_033349	RAI2	retinoic acid induced 2	chrX	-5.08465	0.758442	Down
NM_203365	RAPH1	Ras association (RalGDS/AF-6) and pleckstrin homology domains 1	chr2	-4.95852	1.22006	Down
NM_206963	RARRES1	retinoic acid receptor responder (tazarotene induced) 1	chr3	-2.74918	3.03013	Down
NM_001193520	RASAL1	RAS protein activator like 1 (GAP1 like)	chr12	-3.57515	2.79598	Down
NM_001193521	RASAL1	RAS protein activator like 1 (GAP1 like)	chr12	-3.57515	2.79598	Down
NM_004658	RASAL1	RAS protein activator like 1 (GAP1 like)	chr12	-3.57515	2.79598	Down
NM_177532	RASSF6	Ras association (RalGDS/AF-6) domain family member 6	chr4	-3.33998	2.75157	Down
NM_201431	RASSF6	Ras association (RalGDS/AF-6) domain family member 6	chr4	-3.33998	2.75157	Down
NM_005611	RBL2	retinoblastoma-like 2 (p130)	chr16	1.2321	1.14311	Down
NM_001003792	RBMS3	RNA binding motif, single stranded interacting protein	chr3	-3.48453	2.77247	Down
NM_001003793	RBMS3	RNA binding motif, single stranded interacting protein	chr3	-3.48453	2.77247	Down
NM_001177711	RBMS3	RNA binding motif, single stranded interacting protein	chr3	-3.48453	2.77247	Down
NM_001251985	RCAN3	RCAN family member 3	chr1	-1.00862	1.40474	Down
NM_001163122	RDM1	RAD52 motif 1	chr17	-3.09185	2.67254	Down
NM_032498	RHOXF2	Rhox homeobox family, member 2	chrX	-3.43737	3.45685	Down
NM_001099685	RHOXF2B	Rhox homeobox family, member 2B	chrX	-3.43737	3.45685	Down
NM_144968	RIBC1	RIB43A domain with coiled-coils 1	chrX	-4.93459	2.09883	Down
NM_173642	RIMKLA	ribosomal modification protein rimK-like family member A	chr1	-2.9921	4.07001	Down
NM_134441	RLN2	relaxin 2	chr9	-3.50147	2.71367	Down
NM_002935	RNASE3	ribonuclease, RNase A family, 3 (eosinophil cationic protein)	chr14	-2.61786	0.00637026	Down
NM_018363	RNLS	renalase, FAD-dependent amine oxidase	chr10	-4.62508	1.44832	Down
NM_001199355	RPL17-C18ORI	RPL17-C18orf32 readthrough	chr18	1.0868	1.31567	Down
NM_001199356	RPL17-C18ORI	RPL17-C18orf32 readthrough	chr18	1.07893	1.3127	Down
NM_000983	RPL22	ribosomal protein L22 pseudogene 11; ribosomal protein L22	chr1	1.03	1.16812	Down
NM_000987	RPL26	ribosomal protein L26 pseudogene 33; ribosomal protein L26; ribosomal protein L26 pseudogene 16; ribosomal protein L26 pseudogene 19; ribosomal protein L26 pseudogene 6	chr17	0.501229	1.4295	Down
NM_000967	RPL3	ribosomal protein L3; similar to 60S ribosomal protein L3 (L4)	chr22	2.91192	0.905696	Down
NM_001033853	RPL3	ribosomal protein L3; similar to 60S ribosomal protein L3 (L4)	chr22	2.84777	0.904441	Down
NM_033625	RPL34	ribosomal protein L34	chr4	-0.377175	1.60439	Down
NM_000995	RPL34	ribosomal protein L34	chr4	-0.38211	1.61331	Down
NM_000998	RPL37A	ribosomal protein L37a	chr2	2.1456	0.847576	Down
NM_001024662	RPL6	ribosomal protein L6 pseudogene 27; ribosomal protein L6 pseudogene 19; ribosomal protein L6; ribosomal protein L6 pseudogene 10	chr12	1.92649	1.03436	Down

NM_000970	RPL6	ribosomal protein L6 pseudogene 27; ribosomal protein L6 pseudogene 19; ribosomal protein L6; ribosomal protein L6 pseudogene 10	chr12	1.92491	1.03579	Down
NM_000971	RPL7	ribosomal protein L7 pseudogene 26; ribosomal protein L7 pseudogene 16; ribosomal protein L7; ribosomal protein L7 pseudogene 32; ribosomal protein L7 pseudogene 23; ribosomal protein L7 pseudogene 24; ribosomal protein L7 pseudogene 20	chr8	0.762432	1.21402	Down
NM_053275	RPLP0	ribosomal protein, large, P0 pseudogene 2; ribosomal protein, large, P0 pseudogene 3; ribosomal protein, large, P0 pseudogene 6; ribosomal protein, large, P0	chr12	2.50231	0.854574	Down
NM_001002	RPLP0	ribosomal protein, large, P0 pseudogene 2; ribosomal protein, large, P0 pseudogene 3; ribosomal protein, large, P0 pseudogene 6; ribosomal protein, large, P0	chr12	2.50228	0.854521	Down
NM_001030	RPS27	ribosomal protein S27 pseudogene 29; ribosomal protein S27 pseudogene 9; ribosomal protein S27 pseudogene 23; ribosomal protein S27 pseudogene 13; ribosomal protein S27; ribosomal protein S27 pseudogene 21; ribosomal protein S27 pseudogene 7; ribosomal protein S27 pseudogene 6; ribosomal protein S27 pseudogene 19	chr1	2.23488	0.975008	Down
NM_001009	RPS5	ribosomal protein S5	chr19	2.1474	0.835184	Down
NM_001011	RPS7	ribosomal protein S7; ribosomal protein S7 pseudogene 11; ribosomal protein S7 pseudogene 4; ribosomal protein S7 pseudogene 10	chr2	0.348769	1.38346	Down
NM_001012	RPS8	ribosomal protein S8; ribosomal protein S8 pseudogene 8; ribosomal protein S8 pseudogene 10	chr1	2.03561	0.983243	Down
NM_002295	RPSA	ribosomal protein SA pseudogene 9; ribosomal protein SA pseudogene 8; ribosomal protein SA pseudogene 58; ribosomal protein SA pseudogene 19; ribosomal protein SA pseudogene 18; ribosomal protein SA; ribosomal protein SA pseudogene 15; ribosomal protein SA pseudogene 61; ribosomal protein SA pseudogene 29; ribosomal protein SA pseudogene 12	chr3	2.03855	1.04254	Down
NM_001012321	RPSA	ribosomal protein SA pseudogene 9; ribosomal protein SA pseudogene 8; ribosomal protein SA pseudogene 58; ribosomal protein SA pseudogene 19; ribosomal protein SA pseudogene 18; ribosomal protein SA; ribosomal protein SA pseudogene 15; ribosomal protein SA pseudogene 61; ribosomal protein SA pseudogene 29; ribosomal protein SA pseudogene 12	chr3	2.03846	1.04266	Down
NM_080860	RSPH1	radial spoke head 1 homolog (Chlamydomonas)	chr21	-4.2895	2.19218	Down
NM_178568	RTN4RL1	reticulon 4 receptor-like 1	chr17	-4.57869	0.0356964	Down
NM_005620	S100A11	S100 calcium binding protein A11; S100 calcium binding protein A11 pseudogene	chr1	3.12789	0.42973	Down
NM_014139	SCN11A	sodium channel, voltage-gated, type XI, alpha subunit	chr3	-1.43296	2.86591	Down
NM_006843	SDS	serine dehydratase	chr12	-3.40264	2.9161	Down
NR_039978	SEC24B-AS1	SEC24B antisense RNA 1	chr4	-4.37605	2.13374	Down
NM_207366	SEPT14	septin 14	chr7	-2.7104	2.78653	Down
NM_001174072	SERINC5	serine incorporator 5	chr5	-0.795155	1.42651	Down
NM_012433	SF3B1	splicing factor 3b, subunit 1, 155kDa	chr2	1.40054	1.08428	Down
NM_152550	SH3RF2	SH3 domain containing ring finger 2	chr5	-4.12348	2.56527	Down
NM_016148	SHANK1	SH3 and multiple ankyrin repeat domains 1	chr19	-3.6249	2.31781	Down
NM_001031807	SKOR1	LBXCOR1 homolog (mouse)	chr15	-3.16487	2.31704	Down
NM_001134771	SLC12A5	solute carrier family 12 (potassium-chloride transporter), member 5	chr20	-3.16463	4.55844	Down
NM_022444	SLC13A1	solute carrier family 13 (sodium/sulfate symporters), member 1	chr7	-4.57879	1.60829	Down
NM_001242757	SLC22A31	solute carrier family 22, member 31	chr16	-3.23899	3.96951	Down
NM_030777	SLC2A10	solute carrier family 2 (facilitated glucose transporter), member 10	chr20	-3.74268	2.91935	Down
NM_001134658	SLC35G1	transmembrane protein 20	chr10	-5.04819	1.31911	Down
NM_153226	SLC35G1	transmembrane protein 20	chr10	-5.04819	1.31911	Down

NM_030674	SLC38A1	solute carrier family 38, member 1	chr12	0.552855	1.33726	Down
NM_001077484	SLC38A1	solute carrier family 38, member 1	chr12	0.548706	1.3349	Down
NM_001142405	SLMO1	slowmo homolog 1 (Drosophila)	chr18	-3.24399	1.78958	Down
NM_006553	SLMO1	slowmo homolog 1 (Drosophila)	chr18	-3.24399	1.78958	Down
NR_002922	SNORA13	small nucleolar RNA, H/ACA box 13	chr5	-1.99014	2.56189	Down
NR_002959	SNORA18	small nucleolar RNA, H/ACA box 18	chr11	-2.73717	2.34077	Down
NR_002961	SNORA22	small nucleolar RNA, H/ACA box 22	chr7	-3.14164	2.61171	Down
NR_003016	SNORA26	small nucleolar RNA, H/ACA box 26	chr4	-2.06268	4.12537	Down
NR_002950	SNORA2A	small nucleolar RNA, H/ACA box 2A; small nucleolar RNA, H/ACA box 2B	chr12	-1.39823	0.277259	Down
NR_003015	SNORA53	small nucleolar RNA, H/ACA box 53	chr12	-5.36155	0.163116	Down
NR_002984	SNORA56	small nucleolar RNA, H/ACA box 56	chrX	-4.81161	1.68325	Down
NR_003019	SNORA77	small nucleolar RNA, H/ACA box 77	chr1	-4.98176	1.56656	Down
NR_002992	SNORA7B	small nucleolar RNA, H/ACA box 7A; small nucleolar RNA, H/ACA box 7B; ribosomal protein L32	chr3	-4.93254	1.853	Down
NR_004054	SNORD103A	small nucleolar RNA, C/D box 103A; small nucleolar RNA, C/D box 85	chr1	-2.7503	2.70415	Down
NR_003694	SNORD11B	small nucleolar RNA, C/D box 11B	chr2	-3.59402	1.77848	Down
NR_002440	SNORD16	small nucleolar RNA, C/D box 16	chr15	-5.34265	1.6761	Down
NR_002587	SNORD2	small nucleolar RNA, C/D box 2	chr3	-3.85573	3.45251	Down
NR_002563	SNORD27	small nucleolar RNA, C/D box 27	chr11	-2.76121	3.60821	Down
NR_002562	SNORD28	small nucleolar RNA, C/D box 28	chr11	-3.20596	2.12265	Down
NR_000018	SNORD35A	small nucleolar RNA, C/D box 35B; small nucleolar RNA, C/D box 35A	chr19	-3.71277	2.11158	Down
NR_000013	SNORD42B	small nucleolar RNA, C/D box 42B	chr17	-3.06324	3.79063	Down
NR_002437	SNORD54	small nucleolar RNA, C/D box 54	chr8	-3.72361	2.23618	Down
NR_002571	SNORD58A	U58 small nucleolar RNA; small nucleolar RNA, C/D box 58C; small nucleolar RNA, C/D box 58A; small nucleolar RNA, C/D box 58B	chr18	-3.961	3.63273	Down
NR_002736	SNORD60	small nucleolar RNA, C/D box 60	chr16	-1.98151	1.62718	Down
NR_002914	SNORD62A	small nucleolar RNA, C/D box 62A; small nucleolar RNA, C/D box 62B	chr9	-4.77567	1.65321	Down
NR_003050	SNORD62B	small nucleolar RNA, C/D box 62A; small nucleolar RNA, C/D box 62B	chr9	-4.77567	1.65321	Down
NR_003939	SNORD79	small nucleolar RNA, C/D box 79	chr1	-4.54072	2.13574	Down
NR_000028	SNORD83B	small nucleolar RNA, C/D box 83A; small nucleolar RNA, C/D box 83B	chr22	-4.04413	2.98575	Down
NR_002592	SNORD96A	small nucleolar RNA, C/D box 96B; small nucleolar RNA, C/D box 96A	chr5	-4.45806	1.69275	Down
NM_178010	SOX5	SRY (sex determining region Y)-box 5	chr12	-2.03379	4.06758	Down
NM_206996	SPAG17	sperm associated antigen 17	chr1	-3.8425	3.03921	Down
NM_198546	SPATA21	spermatogenesis associated 21	chr1	-3.91669	2.73686	Down
NR_045180	SPG20OS	SPG20 opposite strand	chr13	-3.03955	6.0791	Down
NM_001080525	SPINK8	serine peptidase inhibitor, Kazal type 8 (putative)	chr3	-4.42757	2.67425	Down
NM_001244950	SPOCK2	sparc/osteonectin, cwcv and kazal-like domains proteoglycan (testican) 2	chr10	1.97483	0.931337	Down
NM_014767	SPOCK2	sparc/osteonectin, cwcv and kazal-like domains proteoglycan (testican) 2	chr10	1.97455	0.930667	Down
NR_033789	SPRNP1	shadow of prion protein homolog (zebrafish) pseudogene 1	chr10	-3.8773	2.0012	Down
NM_018327	SPTLC3	serine palmitoyltransferase, long chain base subunit 3	chr20	-3.32209	2.97137	Down
NM_080823	SRMS	src-related kinase lacking C-terminal regulatory tyrosine and N-terminal myristylation sites	chr20	-2.94092	3.08538	Down
NM_001050	SSTR2	somatostatin receptor 2	chr17	-2.63431	3.62637	Down
NM_207342	STEAP1B	similar to Six transmembrane epithelial antigen of prostate	chr7	-4.7263	1.96159	Down
NM_001040665	STEAP2	six transmembrane epithelial antigen of the prostate 2	chr7	-4.97704	1.29792	Down
NM_001244944	STEAP2	six transmembrane epithelial antigen of the prostate 2	chr7	-4.97704	1.29792	Down
NM_152999	STEAP2	six transmembrane epithelial antigen of the prostate 2	chr7	-4.97704	1.29792	Down
NM_004197	STK19	serine/threonine kinase 19	chr6	-5.16649	1.9123	Down
NM_032454	STK19	serine/threonine kinase 19	chr6	-5.16649	1.9123	Down
NM_033025	SYDE1	synapse defective 1, Rho GTPase, homolog 1 (C. elegans)	chr19	-2.91222	2.51588	Down
NM_001114133	SYNPO2L	synaptopodin 2-like	chr10	-3.2809	4.14722	Down

NM_031912	SYT15	synaptotagmin XV	chr10	-2.90577	1.62238	Down
NM_003564	TAGLN2	transgelin 2	chr1	3.62347	0.305031	Down
NM_003190	TAPBP	TAP binding protein (tapasin)	chr6	2.61399	0.793616	Down
NR_002924	TBC1D3P1-DH	DEAH (Asp-Glu-Ala-His) box polypeptide 40 pseudogene	chr17	-2.38586	1.69801	Down
NM_001128596	TC2N	tandem C2 domains, nuclear	chr14	-0.380333	1.55368	Down
NM_152332	TC2N	tandem C2 domains, nuclear	chr14	-0.411949	1.54975	Down
NM_001128595	TC2N	tandem C2 domains, nuclear	chr14	-0.413358	1.5501	Down
NM_004918	TCL1B	T-cell leukemia/lymphoma 1B	chr14	-4.28376	0.0187825	Down
NM_031898	TEKT3	tektin 3	chr17	-2.12097	4.24194	Down
NR_033777	TEX21P	similar to testis expressed gene 21	chr14	-4.70535	0.187495	Down
NM_001206840	TGOLN2	trans-golgi network protein 2	chr2	1.54937	0.97607	Down
NM_001206841	TGOLN2	trans-golgi network protein 2	chr2	1.54826	0.975007	Down
NM_006464	TGOLN2	trans-golgi network protein 2	chr2	1.54483	0.974202	Down
NM_001206844	TGOLN2	trans-golgi network protein 2	chr2	1.53559	0.972527	Down
NM_001164685	THEMIS	thymocyte selection pathway associated	chr6	-1.38755	1.59177	Down
NM_003251	THRSP	thyroid hormone responsive (SPOT14 homolog, rat)	chr11	-2.30641	1.81637	Down
NM_001146726	TIMD4	T-cell immunoglobulin and mucin domain containing 4	chr5	-2.68473	1.94207	Down
NM_138379	TIMD4	T-cell immunoglobulin and mucin domain containing 4	chr5	-2.68473	1.94207	Down
NM_006289	TLN1	talin 1	chr9	3.26132	0.289714	Down
NM_153345	TMEM139	transmembrane protein 139	chr7	-4.46982	1.9466	Down
NM_001123228	TMEM14E	transmembrane protein 14E	chr3	-3.37301	2.82702	Down
NM_022484	TMEM168	transmembrane protein 168	chr7	-1.95419	1.55826	Down
NM_001098844	TMEM236	family with sequence similarity 23, member A	chr10	-3.53072	4.54224	Down
NM_030770	TMPRSS5	transmembrane protease, serine 5	chr11	-5.27238	0.822326	Down
NM_021103	TMSB10	thymosin beta 10	chr2	2.85399	0.735609	Down
NM_181783	TMTC3	transmembrane and tetratricopeptide repeat containing 3	chr12	-3.47236	2.18321	Down
NM_002160	TNC	tenascin C	chr9	-5.44309	0.52833	Down
NM_001204458	TNFRSF19	tumor necrosis factor receptor superfamily, member 19	chr13	-3.56473	3.03871	Down
NM_148957	TNFRSF19	tumor necrosis factor receptor superfamily, member 19	chr13	-3.56473	3.03871	Down
NM_033012	TNFSF11	tumor necrosis factor (ligand) superfamily, member 11	chr13	-5.36702	0.402884	Down
NM_003280	TNNC1	troponin C type 1 (slow)	chr3	-3.0372	3.56592	Down
NM_032865	TNS4	tensin 4	chr17	-4.84002	1.18035	Down
NM_001114979	TP63	tumor protein p63	chr3	-2.43137	2.67177	Down
NM_006670	TPBG	trophoblast glycoprotein	chr6	-2.595	1.86057	Down
NM_001166392	TPBG	trophoblast glycoprotein	chr6	-2.60897	1.88852	Down
NR_027338	TPI1P3	triosephosphate isomerase 1 pseudogene	chr6	-2.44314	4.88627	Down
NM_003295	TPT1	similar to tumor protein, translationally-controlled 1; tumor protein, translationally-controlled 1	chr13	3.03764	0.717623	Down
NR_034111	TRAF3IP2-AS1	TRAF3IP2 antisense RNA 1	chr6	-5.10922	1.01772	Down
NM_001031712	TRMT11	tRNA methyltransferase 11 homolog (S. cerevisiae)	chr6	-3.63882	2.2988	Down
NM_016157	TRO	trophinin	chrX	-3.10036	2.78107	Down
NM_177556	TRO	trophinin	chrX	-3.10036	2.78107	Down
NM_007332	TRPA1	transient receptor potential cation channel, subfamily A, member 1	chr8	-4.05225	2.66643	Down
NM_052933	TSGA13	testis specific, 13	chr7	-2.84689	2.62006	Down
NM_031945	TSPAN10	tetraspanin 10	chr17	-3.79158	1.9173	Down
NM_001042601	TTC14	tetratricopeptide repeat domain 14	chr3	-1.01792	1.59693	Down
NM_001029964	TTLL13	tubulin tyrosine ligase-like family, member 13	chr15	-5.08508	0.642159	Down
NR_001542	TTTY10	testis-specific transcript, Y-linked 10	chrY	-3.59243	2.74149	Down
NM_001005367	TTYH1	tweety homolog 1 (Drosophila)	chr19	-4.97853	0.820382	Down
NM_006009	TUBA1A	tubulin, alpha 1a	chr12	2.06693	0.310927	Down
NM_080386	TUBA3D	tubulin, alpha 3d; tubulin, alpha 3c	chr2	-4.12974	2.08211	Down
NM_013438	UBQLN1	ubiquilin 1	chr9	0.660522	0.137587	Down
NM_053067	UBQLN1	ubiquilin 1	chr9	0.658486	0.134565	Down

NR_031764	UGT3A2	UDP glycosyltransferase 3 family, polypeptide A2	chr5	-2.25418	1.71191	Down
NM_024518	ULBP3	UL16 binding protein 3	chr6	-2.44448	2.9921	Down
NR_003190	USP32P1	TL132 pseudogene	chr17	-4.66797	1.36206	Down
NM_020633	VN1R1	vomeronasal 1 receptor 1	chr19	-5.28403	1.39995	Down
NM_001077621	VPS37D	vacuolar protein sorting 37 homolog D (S. cerevisiae)	chr7	-3.41338	3.13884	Down
NM_025145	WDR96	chromosome 10 open reading frame 79	chr10	-3.6232	3.27294	Down
NM_016087	WNT16	wingless-type MMTV integration site family, member 16	chr7	-4.77633	0.440155	Down
NM_001168278	WWTR1	WW domain containing transcription regulator 1	chr3	-3.96391	2.20012	Down
NM_003404	YWHAB	tyrosine 3-monooxygenase/tryptophan 5-monooxygenase activation protein, beta polypeptide	chr20	2.13241	0.741841	Down
NM_139323	YWHAB	tyrosine 3-monooxygenase/tryptophan 5-monooxygenase activation protein, beta polypeptide	chr20	2.13241	0.741841	Down
NM_033390	ZC3H12C	zinc finger CCCH-type containing 12C	chr11	-4.25885	2.45704	Down
NM_004926	ZFP36L1	zinc finger protein 36, C3H type-like 1	chr14	2.16951	0.898177	Down
NM_001244701	ZFP36L1	zinc finger protein 36, C3H type-like 1	chr14	2.16835	0.897177	Down
NM_152283	ZFP62	zinc finger protein 62 homolog (mouse)	chr5	-2.27541	1.65077	Down
NM_001172638	ZFP62	zinc finger protein 62 homolog (mouse)	chr5	-2.29549	1.68181	Down
NM_173531	ZNF100	zinc finger protein 100	chr19	-2.90028	1.63667	Down
NM_001032374	ZNF226	zinc finger protein 226	chr19	-4.23506	1.95069	Down
NM_001146220	ZNF226	zinc finger protein 226	chr19	-4.23506	1.95069	Down
NM_015919	ZNF226	zinc finger protein 226	chr19	-4.34528	2.00683	Down
NM_001243242	ZNF323	zinc finger protein 323	chr6	-2.90033	3.60967	Down
NM_001243244	ZNF323	zinc finger protein 323	chr6	-2.90033	3.60967	Down
NM_199441	ZNF334	zinc finger protein 334	chr20	-2.45912	2.68808	Down
NM_001164309	ZNF415	zinc finger protein 415	chr19	-4.19723	2.30355	Down
NM_001136038	ZNF415	zinc finger protein 415	chr19	-4.20149	2.29503	Down
NM_006635	ZNF460	zinc finger protein 460	chr19	-3.85679	2.38293	Down
NM_001101419	ZNF541	zinc finger protein 541	chr19	-3.56883	2.27288	Down
NM_001042415	ZNF596	zinc finger protein 596	chr8	-4.2776	1.66112	Down
NM_173539	ZNF596	zinc finger protein 596	chr8	-4.51254	1.52194	Down
NM_201269	ZNF644	zinc finger protein 644	chr1	-1.70865	1.78956	Down
NM_001033723	ZNF704	zinc finger protein 704	chr8	-2.95017	3.84107	Down
NM_001005851	ZNF780B	zinc finger protein 780B	chr19	-1.52132	1.79334	Down
NM_152605	ZNF781	zinc finger protein 781	chr19	-2.93944	2.76082	Down
NM_207341	ZP1	zona pellucida glycoprotein 1 (sperm receptor)	chr11	-4.44673	1.7687	Down
NM_001004339	ZYG11A	zyg-11 homolog A (C. elegans)	chr1	-1.8051	2.05232	Down

*AG value: Average gene expression

*AD value: Average difference of gene expression
